# Supplementary material for: What Is So Special about Quantum Clicks?
Source: Entropy (Basel). 2020 May 28;22(6):602. doi: 10.3390/e22060602 (PMC7517140; doi:10.3390/e22060602)
Supplement: Supplementary file 1 [file entropy-22-00602-s001.pdf]

Supplemental Material: What Is so Special About Quantum Clicks?

Karl Svozil*
Institute for Theoretical Physics, Vienna University of Technology, Wiedner Hauptstrasse 8-10/136, 1040 Vienna, Austria
(Dated: May 26, 2020)

This supplement contains mostly code interpretable by Fukuda's *cddlib package cddlib-094h* for evaluating hull problems in quantum physical configurations. It also contains some corresponding quantum mechanical calculations.

* svozil@tuwien.ac.at; http://tph.tuwien.ac.at/~svozil

CONTENTS

A. The cddlib package 2
B. Trivial examples 3
1. One observable 3
2. Two observables 3
3. Bounds on the (joint) probabilities and expectations of three observables 4
C. 2 observers, 2 measurement configurations per observer 5
1. Bell-Wigner-Fine case: probabilities for 2 observers, 2 measurement configurations per observer 6
2. Clauser-Horne-Shimony-Holt case: expectation values for 2 observers, 2 measurement configurations per observer 7
3. Beyond the Clauser-Horne-Shimony-Holt case: 2 observers, 3 measurement configurations per observer 8
D. Pentagon logic 10
E. Probabilities but no joint probabilities 10
F. Joint Expectations on all atoms 11

I. House/pentagon/pentagram gadget 15
1. Bub-Stairs inequality 15
2. Klyachko-Can-Biniciogolu-Shumovsky inequalities 16
A. Two intertwined pentagon logics forming a Specker Käfer (bug) or cat's cradle logic 17
1. Probabilities on the Specker bug logic 17
2. Hull calculation for the probabilities on the Specker bug logic 18
3. Hull calculation for the expectations on the Specker bug logic 20
4. Extended Specker bug logic 20
B. Two intertwined Specker bug logics 21
1. Hull calculation for the contexual inequalities corresponding to the Cabello, Estebaranz and García-Alcaine logic 22
2. Hull calculation for the contexual inequalities corresponding to the pentagon logic 31
3. Hull calculation for the contexual inequalities corresponding to Specker bug logics 32
4. Min-max calculation for the quantum bounds of two-two-state particles 34
5. Min-max calculation for the quantum bounds of two three-state particles 41
6. Min-max calculation for two four-state particles 46

References 50

A. The cddlib package

Fukuda's *cddlib package cddlib-094h* can be obtained from the package homepage [1]. Installation on Unix-type operating systems is with *gcc*; the free library for arbitrary precision arithmetic *GMP* (currently 6.1.2) [2], must be installed first.

In its elementary form of the *V-representation*, *cddlib* takes in the k vertices $|\mathbf{v}_1\rangle, \ldots, |\mathbf{v}_k\rangle$ of a convex polytope in an m-dimensional vector space as follows (note that all rows of vector components start with "1"):

```
V-representation
begin
k   m+1   numbertype
1  v_11  ...  v_1m
...............
1  v_k1  ...  v_km
end
```

cddlib responds with the faces (boundaries of halfspaces), as encoded by n inequalities $\mathbf{A}|\mathbf{x}\rangle \leq |\mathbf{b}\rangle$ in the *H-representation* as follows:

```
H-representation
begin
n       m+1   numbertype
b      -A
end
```

Comments appear after an asterisk.

B. Trivial examples

1. One observable

The case of a single variable has two extreme cases: false$\equiv 0$ and true$\equiv 1$, resulting in $0 \le p_1 \le 1$:

```
* one variable
*
V-representation
begin
2   2   integer
1   0
1   1
end

~~~~~~ cddlib response

H-representation
begin
 2 2 real
  1 -1
  0  1
end
```

2. Two observables

The case of two variables p_1 and p_2, and a joint variable p_{12}, result in

$$p_1 + p_2 - p_{12} \le 1, \tag{1}$$

$$-p_1 + p_{12} \le 0, \tag{2}$$

$$-p_2 + p_{12} \le 0, \tag{3}$$

$$-p_{12} \le 0, \tag{4}$$

and thus $0 \le p_{12} \le p_1, p_2$.

```
* two variables: p1, p2, p12=p1*p2
*
V-representation
begin
4   4   integer
1   0   0   0
1   0   1   0
1   1   0   0
1   1   1   1
end

~~~~~~ cddlib response

H-representation
begin
 4 4 real
  1 -1 -1  1
  0  1  0 -1
  0  0  1 -1
  0  0  0  1
end
```

For dichotomic expectation values ± 1,

```
* two expectation values: E1, E2, E12=E1*E2
*
V-representation
begin
4    4    integer
1    -1    -1    1
1    -1    1    -1
1    1    -1    -1
1    1    1    1
end

~~~~~~ cddlib response

H-representation
begin
 4 4 real
  1 -1 -1  1
  1  1 -1 -1
  1 -1  1 -1
  1  1  1  1
end
```

3. Bounds on the (joint) probabilities and expectations of three observables

```
* four joint expectations:
* p1, p2, p3,
* p12=p1*p2, p13=p1*p3, p23=p2*p3,
* p123=p1*p2*p3
V-representation
begin
8    8    integer
1        0    0    0    0    0    0    0
1        0    0    1    0    0    0    0
1        0    1    0    0    0    0    0
1        0    1    1    0    0    1    0
1        1    0    0    0    0    0    0
1        1    0    1    0    1    0    0
1        1    1    0    1    0    0    0
1        1    1    1    1    1    1    1
end

~~~~~~ cddlib response

H-representation
begin
 8 8 real
  1 -1 -1 -1  1  1  1 -1
  0  1  0  0 -1 -1  0  1
  0  0  1  0 -1  0 -1  1
  0  0  0  1  0 -1 -1  1
  0  0  0  0  1  0  0 -1
  0  0  0  0  0  1  0 -1
  0  0  0  0  0  0  1 -1
  0  0  0  0  0  0  0  1
end
```

If single observable expectations are set to zero by assumption (axiom) and are not-enumerated, the table of expectation values may be redundand.

The case of three expectation value observables E_1, E_2 and E_3 (which are not explicitly enumerated), as well as all joint expectations E_{12}, E_{13}, E_{23}, and E_{123}, result in

$$
\begin{aligned}
-E_{12}-E_{13}-E_{23} &\leq 1 \qquad (5)\\
-E_{123} &\leq 1, \qquad (6)\\
E_{123} &\leq 1, \qquad (7)\\
-E_{12}+E_{13}+E_{23} &\leq 1, \qquad (8)\\
E_{12}-E_{13}+E_{23} &\leq 1, \qquad (9)\\
E_{12}+E_{13}-E_{23} &\leq 1. \qquad (10)
\end{aligned}
$$

```
* four joint expectations:
* [E1, E2, E3, not explicitly enumerated]
* E12=E1*E2, E13=E1*E3, E23=E2*E3,
* E123=E1*E2*E3
V-representation
begin
8   5    integer
1    1    1    1    1
1    1   -1   -1   -1
1   -1    1   -1   -1
1   -1   -1    1    1
1   -1   -1    1   -1
1   -1    1   -1    1
1    1   -1   -1    1
1    1    1    1   -1
end

~~~~~~ cddlib response

H-representation
begin
 6 5 real
  1  1  1  1  0
  1  0  0  0  1
  1  0  0  0 -1
  1  1 -1 -1  0
  1 -1  1 -1  0
  1 -1 -1  1  0
end
```

C. 2 observers, 2 measurement configurations per observer

From a quantum physical standpoint the first relevant case is that of 2 observers and 2 measurement configurations per observer.

1. Bell-Wigner-Fine case: probabilities for 2 observers, 2 measurement configurations per observer

The case of four probabilities p_1, p_2, p_3 and p_4, as well as four joint probabilities p_{13}, p_{14}, p_{23}, and p_{24} result in

$$-p_{14} \le 0 \tag{11}$$
$$-p_{24} \le 0 \tag{12}$$
$$+p_1 + p_4 - p_{13} - p_{14} + p_{23} - p_{24} \le 1 \tag{13}$$
$$+p_2 + p_4 + p_{13} - p_{14} - p_{23} - p_{24} \le 1 \tag{14}$$
$$+p_2 + p_3 - p_{13} + p_{14} - p_{23} - p_{24} \le 1 \tag{15}$$
$$+p_1 + p_3 - p_{13} - p_{14} - p_{23} + p_{24} \le 1 \tag{16}$$
$$-p_{13} \le 0 \tag{17}$$
$$-p_{23} \le 0 \tag{18}$$
$$-p_1 - p_4 + p_{13} + p_{14} - p_{23} + p_{24} \le 0 \tag{19}$$
$$-p_2 - p_4 - p_{13} + p_{14} + p_{23} + p_{24} \le 0 \tag{20}$$
$$-p_2 - p_3 + p_{13} - p_{14} + p_{23} + p_{24} \le 0 \tag{21}$$
$$-p_1 - p_3 + p_{13} + p_{14} + p_{23} - p_{24} \le 0 \tag{22}$$
$$-p_1 + p_{14} \le 0 \tag{23}$$
$$-p_2 + p_{24} \le 0 \tag{24}$$
$$-p_3 + p_{23} \le 0 \tag{25}$$
$$-p_3 + p_{13} \le 0 \tag{26}$$
$$-p_1 + p_{13} \le 0 \tag{27}$$
$$-p_2 + p_{23} \le 0 \tag{28}$$
$$-p_4 + p_{24} \le 0 \tag{29}$$
$$-p_4 + p_{14} \le 0 \tag{30}$$
$$+p_2 + p_4 - p_{24} \le 1 \tag{31}$$
$$+p_1 + p_4 - p_{14} \le 1 \tag{32}$$
$$+p_2 + p_3 - p_{23} \le 1 \tag{33}$$
$$+p_1 + p_3 - p_{13} \le 1. \tag{34}$$

```
* eight variables: p1, p2, p3, p4,
* p13, p14, p23, p24
*
V-representation
begin
16   9   integer
1    0    0    0    0    0    0    0    0
1    0    0    0    1    0    0    0    0
1    0    0    1    0    0    0    0    0
1    0    0    1    1    0    0    0    0
1    0    1    0    0    0    0    0    0
1    0    1    0    1    0    0    0    1
1    0    1    1    0    0    0    1    0
1    0    1    1    1    0    0    1    1
1    1    0    0    0    0    0    0    0
1    1    0    0    1    0    1    0    0
1    1    0    1    0    1    0    0    0
1    1    0    1    1    1    1    0    0
1    1    1    0    0    0    0    0    0
1    1    1    0    1    0    1    0    1
1    1    1    1    0    1    0    1    0
1    1    1    1    1    1    1    1    1
end

~~~~~~ cddlib response
```

```
H-representation
begin
 24 9 real
  0  0  0  0  0  0  1  0  0
  0  0  0  0  0  0  0  0  1
  1 -1  0  0 -1  1  1 -1  1
  1  0 -1  0 -1 -1  1  1  1
  1  0 -1 -1  0  1 -1  1  1
  1 -1  0 -1  0  1  1  1 -1
  0  0  0  0  0  1  0  0  0
  0  0  0  0  0  0  0  1  0
  0  1  0  0  1 -1 -1  1 -1
  0  0  1  0  1  1 -1 -1 -1
  0  0  1  1  0 -1  1 -1 -1
  0  1  0  1  0 -1 -1 -1  1
  0  1  0  0  0  0 -1  0  0
  0  0  1  0  0  0  0  0 -1
  0  0  0  1  0  0  0 -1  0
  0  0  0  1  0 -1  0  0  0
  0  1  0  0  0 -1  0  0  0
  0  0  1  0  0  0  0 -1  0
  0  0  0  0  1  0  0  0 -1
  0  0  0  0  1  0 -1  0  0
  1  0 -1  0 -1  0  0  0  1
  1 -1  0  0 -1  0  1  0  0
  1  0 -1 -1  0  0  0  1  0
  1 -1  0 -1  0  1  0  0  0
end
```

2. *Clauser-Horne-Shimony-Holt case: expectation values for 2 observers, 2 measurement configurations per observer*

The case of four expectation values E_1, E_2, E_3 and E_4 (which are not explicitly enumerated), as well as all joint expectations E_{13}, E_{14}, E_{23}, and E_{24} result in

$$+E_{13}-E_{14}-E_{23}-E_{24}\le 2 \tag{35}$$
$$-E_{24}\le 1 \tag{36}$$
$$-E_{23}\le 1 \tag{37}$$
$$-E_{13}+E_{14}-E_{23}-E_{24}\le 2 \tag{38}$$
$$-E_{14}\le 1 \tag{39}$$
$$-E_{13}-E_{14}+E_{23}-E_{24}\le 2 \tag{40}$$
$$-E_{13}-E_{14}-E_{23}+E_{24}\le 2 \tag{41}$$
$$-E_{13}\le 1 \tag{42}$$
$$-E_{13}+E_{14}+E_{23}+E_{24}\le 2 \tag{43}$$
$$+E_{24}\le 1 \tag{44}$$
$$+E_{23}\le 1 \tag{45}$$
$$+E_{13}-E_{14}+E_{23}+E_{24}\le 2 \tag{46}$$
$$+E_{14}\le 1 \tag{47}$$
$$+E_{13}+E_{14}-E_{23}+E_{24}\le 2 \tag{48}$$
$$+E_{13}+E_{14}+E_{23}-E_{24}\le 2 \tag{49}$$
$$+E_{13}\le 1. \tag{50}$$

```
* four joint expectations:
* E13, E14, E23, E24
```

```
*
V-representation
begin
16    5    integer
1    1    1    1    1
1    1   -1    1   -1
1   -1    1   -1    1
1   -1   -1   -1   -1
1    1    1   -1   -1
1    1   -1   -1    1
1   -1    1    1   -1
1   -1   -1    1    1
1   -1   -1    1    1
1   -1    1    1   -1
1    1   -1   -1    1
1    1    1   -1   -1
1   -1   -1   -1   -1
1   -1    1   -1    1
1    1   -1    1   -1
1    1    1    1    1
end

~~~~~~ cddlib response

H-representation
begin
 16 5 real
  2 -1  1  1  1
  1  0  0  0  1
  1  0  0  1  0
  2  1 -1  1  1
  1  0  1  0  0
  2  1  1 -1  1
  2  1  1  1 -1
  1  1  0  0  0
  2  1 -1 -1 -1
  1  0  0  0 -1
  1  0  0 -1  0
  2 -1  1 -1 -1
  1  0 -1  0  0
  2 -1 -1  1 -1
  2 -1 -1 -1  1
  1 -1  0  0  0
end
```

3. *Beyond the Clauser-Horne-Shimony-Holt case: 2 observers, 3 measurement configurations per observer*

```
* 6  expectations:
* E1, ... , E6
* 9 joint expectations:
* E14, E15, E16, E24, E25, E26, E34, E35, E36
* 1,2,3 on one side
* 4,5,6 on other side
*
V-representation
begin
64   16    integer
1      1    1    1    1    1    1    1    1    1    1    1    1    1    1    1
1      1    1    1    1    1   -1    1    1   -1    1    1   -1    1    1   -1
1      1    1    1    1   -1    1    1   -1    1    1   -1    1    1   -1    1
```

```
1   1   1   1   1  -1  -1   1  -1  -1   1  -1  -1   1  -1  -1
1   1   1   1  -1   1   1  -1   1   1  -1   1   1  -1   1   1
1   1   1   1  -1   1  -1  -1   1  -1  -1   1  -1  -1   1  -1
1   1   1   1  -1  -1   1  -1  -1   1  -1  -1   1  -1  -1   1
1   1   1   1  -1  -1  -1  -1  -1  -1  -1  -1  -1  -1  -1  -1
1   1   1  -1   1   1   1   1   1   1   1   1   1  -1  -1  -1
1   1   1  -1   1   1  -1   1   1  -1   1   1  -1  -1  -1   1
1   1   1  -1   1  -1   1   1  -1   1   1  -1   1  -1   1  -1
1   1   1  -1   1  -1  -1   1  -1  -1   1  -1  -1  -1   1   1
1   1   1  -1  -1   1   1  -1   1   1  -1   1   1   1  -1  -1
1   1   1  -1  -1   1  -1  -1   1  -1  -1   1  -1   1  -1   1
1   1   1  -1  -1  -1   1  -1  -1   1  -1  -1   1   1   1  -1
1   1   1  -1  -1  -1  -1  -1  -1  -1  -1  -1  -1   1   1   1
1   1  -1   1   1   1   1   1   1   1  -1  -1  -1   1   1   1
1   1  -1   1   1   1  -1   1   1  -1  -1  -1   1   1   1  -1
1   1  -1   1   1  -1   1   1  -1   1  -1   1  -1   1  -1   1
1   1  -1   1   1  -1  -1   1  -1  -1  -1   1   1   1  -1  -1
1   1  -1   1  -1   1   1  -1   1   1   1  -1  -1  -1   1   1
1   1  -1   1  -1   1  -1  -1   1  -1   1  -1   1  -1   1  -1
1   1  -1   1  -1  -1   1  -1  -1   1   1   1  -1  -1  -1   1
1   1  -1   1  -1  -1  -1  -1  -1  -1   1   1   1  -1  -1  -1
1   1  -1  -1   1   1   1   1   1   1  -1  -1  -1  -1  -1  -1
1   1  -1  -1   1   1  -1   1   1  -1  -1  -1   1  -1  -1   1
1   1  -1  -1   1  -1   1   1  -1   1  -1   1  -1  -1   1  -1
1   1  -1  -1   1  -1  -1   1  -1  -1  -1   1   1  -1   1   1
1   1  -1  -1  -1   1   1  -1   1   1   1  -1  -1   1  -1  -1
1   1  -1  -1  -1   1  -1  -1   1  -1   1  -1   1   1  -1   1
1   1  -1  -1  -1  -1   1  -1  -1   1   1   1  -1   1   1  -1
1   1  -1  -1  -1  -1  -1  -1  -1  -1   1   1   1   1   1   1
1  -1   1   1   1   1   1  -1  -1  -1   1   1   1   1   1   1
1  -1   1   1   1   1  -1  -1  -1   1   1   1  -1   1   1  -1
1  -1   1   1   1  -1   1  -1   1  -1   1  -1   1   1  -1   1
1  -1   1   1   1  -1  -1  -1   1   1   1  -1  -1   1  -1  -1
1  -1   1   1  -1   1   1   1  -1  -1  -1   1   1  -1   1   1
1  -1   1   1  -1   1  -1   1  -1   1  -1   1  -1  -1   1  -1
1  -1   1   1  -1  -1   1   1   1  -1  -1  -1   1  -1  -1   1
1  -1   1   1  -1  -1  -1   1   1   1  -1  -1  -1  -1  -1  -1
1  -1   1  -1   1   1   1  -1  -1  -1   1   1   1  -1  -1  -1
1  -1   1  -1   1   1  -1  -1  -1   1   1   1  -1  -1  -1   1
1  -1   1  -1   1  -1   1  -1   1  -1   1  -1   1  -1   1  -1
1  -1   1  -1   1  -1  -1  -1   1   1   1  -1  -1  -1   1   1
1  -1   1  -1  -1   1   1   1  -1  -1  -1   1   1   1  -1  -1
1  -1   1  -1  -1   1  -1   1  -1   1  -1   1  -1   1  -1   1
1  -1   1  -1  -1  -1   1   1   1  -1  -1  -1   1   1   1  -1
1  -1   1  -1  -1  -1  -1   1   1   1  -1  -1  -1   1   1   1
1  -1  -1   1   1   1   1  -1  -1  -1  -1  -1  -1   1   1   1
1  -1  -1   1   1   1  -1  -1  -1   1  -1  -1   1   1   1  -1
1  -1  -1   1   1  -1   1  -1   1  -1  -1   1  -1   1  -1   1
1  -1  -1   1   1  -1  -1  -1   1   1  -1   1   1   1  -1  -1
1  -1  -1   1  -1   1   1   1  -1  -1   1  -1  -1  -1   1   1
1  -1  -1   1  -1   1  -1   1  -1   1   1  -1   1  -1   1  -1
1  -1  -1   1  -1  -1   1   1   1  -1   1   1  -1  -1  -1   1
1  -1  -1   1  -1  -1  -1   1   1   1   1   1   1  -1  -1  -1
1  -1  -1  -1   1   1   1  -1  -1  -1  -1  -1  -1  -1  -1  -1
1  -1  -1  -1   1   1  -1  -1  -1   1  -1  -1   1  -1  -1   1
1  -1  -1  -1   1  -1   1  -1   1  -1  -1   1  -1  -1   1  -1
1  -1  -1  -1   1  -1  -1  -1   1   1  -1   1   1  -1   1   1
1  -1  -1  -1  -1   1   1   1  -1  -1   1  -1  -1   1  -1  -1
1  -1  -1  -1  -1   1  -1   1  -1   1   1  -1   1   1  -1   1
1  -1  -1  -1  -1  -1   1   1   1  -1   1   1  -1   1   1  -1
1  -1  -1  -1  -1  -1  -1   1   1   1   1   1   1   1   1   1
end

~~~~~~ cddlib response
```

```
H-representation
begin
 684 16 real
  4  0 -1  1 -1 -1  0  1 -1  0  1  1  1 -1 -1  1
  [...]
  4  1  1  0  1  1  0  1  1  1  1  1 -1  1 -1  0
  [...]
end
```

D. Pentagon logic

E. Probabilities but no joint probabilities

Here is a computation which includes all probabilities but no joint probabilities:

```
* ten probabilities:
* p1 ... p10
*
begin
11  11  integer
1       1    0    0    1    0    1    0    1    0    0
1       1    0    0    0    1    0    0    1    0    0
1       1    0    0    1    0    0    1    0    0    0
1       0    0    1    0    0    1    0    1    0    1
1       0    0    1    0    0    0    1    0    0    1
1       0    0    1    0    0    1    0    0    1    0
1       0    1    0    0    1    0    0    1    0    1
1       0    1    0    0    1    0    0    0    1    0
1       0    1    0    1    0    0    1    0    0    1
1       0    1    0    1    0    1    0    0    1    0
1       0    1    0    1    0    1    0    1    0    1
end

~~~~~~ cddlib response

H-representation
linearity 5  12 13 14 15 16
begin
 16 11 real
  0  0  0  0  0  0  1  0  0  0  0
  0  0  0  0  0  0  0  0  1  0  0
  0 -1  0  0  1  0  0  0  1  0  0
  0  0  0  0  1  0  0  0  0  0  0
  0  1  0  0  0  0  0  0  0  0  0
  1 -1 -1  0  1  0 -1  0  0  0  0
  0  0  1  0  0  0  0  0  0  0  0
  1 -2 -1  0  1  0 -1  0  1  0  0
  0  1  1  0 -1  0  0  0  0  0  0
  0  1  1  0 -1  0  1  0 -1  0  0
  1 -1 -1  0  0  0  0  0  0  0  0
 -1  1  1  1  0  0  0  0  0  0  0
  0 -1 -1  0  1  1  0  0  0  0  0
 -1  1  1  0 -1  0  1  1  0  0  0
  0 -1 -1  0  1  0 -1  0  1  1  0
 -1  2  1  0 -1  0  1  0 -1  0  1
end
```

$$+p_6 \geq 0 \tag{51}$$

$$+p_8 \geq 0 \tag{52}$$

$$-p_1 + p_4 + p_8 \geq 0 \tag{53}$$

$$+p_4 \geq 0 \tag{54}$$

$$+p_1 \geq 0 \tag{55}$$

$$-p_1 - p_2 + p_4 - p_6 \geq -1 \tag{56}$$

$$+p_2 \geq 0 \tag{57}$$

$$-2p_1 - p_2 + p_4 - p_6 + p_8 \geq -1 \tag{58}$$

$$+p_1 + p_2 - p_4 \geq 0 \tag{59}$$

$$+p_1 + p_2 - p_4 + p_6 - p_8 \geq 0 \tag{60}$$

$$-p_1 - p_2 \geq -1 \tag{61}$$

$$+p_1 + p_2 + p_3 \geq 1 \tag{62}$$

$$-p_1 - p_2 + p_4 + p_5 \geq 0 \tag{63}$$

$$+p_1 + p_2 - p_4 + p_6 + p_7 \geq 1 \tag{64}$$

$$-p_1 - p_2 + p_4 - p_6 + p_8 + p_9 \geq 0 \tag{65}$$

$$2p_1 + p_2 - p_4 + p_6 - p_8 + p_{10} \geq 1. \tag{66}$$

F. Joint Expectations on all atoms

This is a full hull computation taking all joint expectations into account:

```
* 45 pair expectations:
* E12 ... E910
*
V-representation
begin
11 46 real
1 -1 -1 1 -1 1 -1 1 -1 -1 1 -1 1 -1 1 -1 1 1 -1 1 -1 1 -1 1 1 -1 1 -1 1 -1 -1 -1 1 -1 1 1 -1 1
    -1 -1 -1 1 1 -1 -1 1
1 -1 -1 -1 1 -1 -1 1 -1 -1 1 1 -1 1 1 -1 1 1 1 -1 1 1 -1 1 1 -1 1 1 -1 1 1 -1 -1 1 -1 -1 1 -1
   1 1 -1 1 1 -1 -1 1
1 -1 -1 1 -1 -1 1 -1 -1 -1 1 -1 1 1 -1 1 1 1 -1 1 1 -1 1 1 1 -1 -1 1 -1 -1 -1 1 -1 1 1 1 -1 1
   1 1 -1 -1 -1 1 1 1
1 1 -1 1 1 -1 1 -1 1 -1 -1 1 1 -1 1 -1 1 -1 -1 -1 1 -1 1 -1 1 1 -1 1 -1 1 -1 -1 1 -1 1 -1 -1 1
    -1 1 -1 1 -1 -1 1 -1
1 1 -1 1 1 1 -1 1 1 -1 -1 1 1 1 -1 1 1 -1 -1 -1 -1 1 -1 -1 1 1 1 -1 1 1 -1 1 -1 1 1 -1 -1 1 1
   -1 -1 -1 1 1 -1 -1
1 1 -1 1 1 -1 1 1 -1 1 -1 1 1 -1 1 1 -1 1 -1 -1 1 -1 -1 1 -1 1 -1 1 1 -1 1 -1 1 1 -1 1 -1 -1 1
    -1 1 -1 1 -1 1 -1
1 -1 1 1 -1 1 1 -1 1 -1 -1 -1 1 -1 -1 1 -1 1 1 -1 1 1 -1 1 -1 -1 1 1 -1 1 -1 -1 -1 1 -1 1 1 -1
    1 -1 -1 1 -1 -1 1 -1
1 -1 1 1 -1 1 1 1 -1 1 -1 -1 1 -1 -1 -1 1 -1 1 -1 1 1 1 -1 1 -1 1 1 1 -1 1 -1 -1 -1 1 -1 1 1
   -1 1 1 -1 1 -1 1 -1
1 -1 1 -1 1 1 -1 1 1 -1 -1 1 -1 -1 1 -1 -1 1 -1 1 1 -1 1 1 -1 -1 -1 1 -1 -1 1 1 -1 1 1 -1 -1 1
    1 -1 -1 -1 1 1 -1 -1
1 -1 1 -1 1 -1 1 1 -1 1 -1 1 -1 1 -1 -1 1 -1 -1 1 -1 1 1 -1 1 -1 1 -1 -1 1 -1 -1 1 1 -1 1 -1
   -1 1 -1 1 -1 1 -1 1 -1
1 -1 1 -1 1 -1 1 -1 1 -1 -1 1 -1 1 -1 1 -1 1 -1 1 -1 1 -1 1 -1 -1 1 -1 1 -1 1 -1 1 -1 1 -1 -1
   1 -1 1 -1 1 -1 -1 1 -1
end

~~~~~~ cddlib response

H-representation
```

```
linearity 35  12 13 14 15 16 17 18 19 20 21 22 23 24 25 26 27 28 29 30 31 32 33 34 35 36 37 38
     39 40 41 42 43 44 45 46
begin
 46 46 real
  1 0 −1 −1 0 0 0 0 0 0 0 0 0 0 0 0 0 0 1 0 0 0 0 0 0 0 0 0 0 0 0 0 0 0 0 0 0 0 0 0 0 0 0 0 0
     0
  1 1 0 0 0 0 0 −1 0 0 0 0 0 0 0 −1 0 0 0 0 0 0 0 0 0 0 0 0 0 0 0 0 0 0 0 0 0 0 0 0 0 0 0 0 0
     0
  1 0 0 −1 0 0 0 −1 0 0 0 0 0 0 0 0 0 0 0 0 0 0 0 0 0 0 0 0 1 0 0 0 0 0 0 0 0 0 0 0 0 0 0 0 0
     0
 −1 −1 0 1 0 0 0 0 0 0 0 0 0 1 0 0 0 0 −1 0 1 0 0 0 0 0 0 0 0 0 0 0 0 0 0 0 0 0 0 0 0 0 0 0 0
    0
  0 −1 −1 0 0 0 0 0 0 0 0 0 0 −1 0 0 0 0 0 0 −1 0 0 0 0 0 0 0 0 0 0 0 0 0 0 0 0 0 0 0 0 0 0 0
     0 0
  0 0 1 1 0 −1 0 1 0 0 0 0 0 0 0 0 0 0 0 0 −1 0 0 0 0 0 0 0 −1 0 0 0 0 0 0 0 0 0 0 0 0 0 0 0 0
      0
  1 1 0 0 0 1 0 0 0 0 0 0 0 1 0 0 0 0 0 0 0 0 0 0 0 0 0 0 0 0 0 0 0 0 0 0 0 0 0 0 0 0 0 0 0 0
  0 0 0 0 0 −1 0 1 0 0 0 0 0 −1 0 1 0 0 0 0 0 0 0 0 0 0 0 0 0 0 0 0 0 0 0 0 0 0 0 0 0 0 0 0 0
     0
  1 0 0 0 0 0 0 0 0 0 0 0 0 −1 0 1 0 0 1 0 −1 0 0 0 0 0 0 0 −1 0 0 0 0 0 0 0 0 0 0 0 0 0 0 0 0
      0
  0 0 0 −1 0 1 0 0 0 0 0 0 0 0 0 0 0 0 −1 0 1 0 0 0 0 0 0 0 0 0 0 0 0 0 0 0 0 0 0 0 0 0 0 0 0
     0
  0 0 1 1 0 0 0 0 0 0 0 0 0 1 0 −1 0 0 0 0 1 0 0 0 0 0 0 0 1 0 0 0 0 0 0 0 0 0 0 0 0 0 0 0 0 0
 −1 −1 0 1 1 0 0 0 0 0 0 0 0 0 0 0 0 0 0 0 0 0 0 0 0 0 0 0 0 0 0 0 0 0 0 0 0 0 0 0 0 0 0 0 0 0
  0 0 −1 −1 0 1 1 0 0 0 0 0 0 0 0 0 0 0 0 0 0 0 0 0 0 0 0 0 0 0 0 0 0 0 0 0 0 0 0 0 0 0 0 0 0
     0
 −1 −1 0 1 0 −1 0 1 1 0 0 0 0 0 0 0 0 0 0 0 0 0 0 0 0 0 0 0 0 0 0 0 0 0 0 0 0 0 0 0 0 0 0 0 0
    0
  1 0 −1 −1 0 1 0 −1 0 1 0 0 0 0 0 0 0 0 0 0 0 0 0 0 0 0 0 0 0 0 0 0 0 0 0 0 0 0 0 0 0 0 0 0 0
      0
  1 1 1 0 0 0 0 0 0 0 1 0 0 0 0 0 0 0 0 0 0 0 0 0 0 0 0 0 0 0 0 0 0 0 0 0 0 0 0 0 0 0 0 0 0 0
 −1 −1 0 1 0 0 0 0 0 0 0 1 0 0 0 0 0 0 0 0 0 0 0 0 0 0 0 0 0 0 0 0 0 0 0 0 0 0 0 0 0 0 0 0 0 0
  0 0 0 −1 0 0 0 0 0 0 0 0 1 0 0 0 0 0 0 0 0 0 0 0 0 0 0 0 0 0 0 0 0 0 0 0 0 0 0 0 0 0 0 0 0 0
  0 0 1 1 0 0 0 0 0 0 0 0 0 1 1 0 0 0 0 0 0 0 0 0 0 0 0 0 0 0 0 0 0 0 0 0 0 0 0 0 0 0 0 0 0 0
  0 0 0 −1 0 0 0 0 0 0 0 0 0 −1 0 1 1 0 0 0 0 0 0 0 0 0 0 0 0 0 0 0 0 0 0 0 0 0 0 0 0 0 0 0 0
     0
  0 1 1 1 0 0 0 0 0 0 0 0 0 1 0 −1 0 1 0 0 0 0 0 0 0 0 0 0 0 0 0 0 0 0 0 0 0 0 0 0 0 0 0 0 0 0
  1 1 0 0 0 0 0 0 0 0 0 0 0 0 0 0 0 0 1 1 0 0 0 0 0 0 0 0 0 0 0 0 0 0 0 0 0 0 0 0 0 0 0 0 0 0
 −1 0 0 0 0 0 0 0 0 0 0 0 0 0 0 0 0 0 −1 0 1 1 0 0 0 0 0 0 0 0 0 0 0 0 0 0 0 0 0 0 0 0 0 0 0 0
  1 0 −1 −1 0 0 0 0 0 0 0 0 0 −1 0 1 0 0 1 0 −1 0 1 0 0 0 0 0 0 0 0 0 0 0 0 0 0 0 0 0 0 0 0 0
     0 0
  0 1 1 1 0 0 0 0 0 0 0 0 0 1 0 −1 0 0 0 0 0 0 0 1 0 0 0 0 0 0 0 0 0 0 0 0 0 0 0 0 0 0 0 0 0 0
  0 0 0 −1 0 0 0 0 0 0 0 0 0 −1 0 1 0 0 0 0 0 0 0 0 1 0 0 0 0 0 0 0 0 0 0 0 0 0 0 0 0 0 0 0 0
     0
  0 −1 0 0 0 0 0 0 0 0 0 0 0 0 0 0 0 0 0 0 0 0 0 0 0 1 0 0 0 0 0 0 0 0 0 0 0 0 0 0 0 0 0 0 0 0
 −1 0 0 0 0 0 0 0 0 0 0 0 0 0 0 0 0 0 −1 0 1 0 0 0 0 0 1 0 0 0 0 0 0 0 0 0 0 0 0 0 0 0 0 0 0 0
  0 0 0 0 0 0 0 0 0 0 0 0 0 0 0 0 0 0 0 0 −1 0 0 0 0 0 0 1 0 0 0 0 0 0 0 0 0 0 0 0 0 0 0 0 0 0
 −1 −1 0 0 0 0 0 0 0 0 0 0 0 0 0 0 0 0 −1 0 1 0 0 0 0 0 0 0 1 1 0 0 0 0 0 0 0 0 0 0 0 0 0 0 0
    0
  0 0 0 1 0 0 0 0 0 0 0 0 0 0 0 0 0 0 0 0 −1 0 0 0 0 0 0 0 −1 0 1 0 0 0 0 0 0 0 0 0 0 0 0 0 0
     0
  1 0 0 0 0 −1 0 0 0 0 0 0 0 −1 0 0 0 0 1 0 −1 0 0 0 0 0 0 0 0 0 0 1 0 0 0 0 0 0 0 0 0 0 0 0 0
      0
  0 0 0 0 0 1 0 0 0 0 0 0 0 1 0 0 0 0 0 0 1 0 0 0 0 0 0 0 0 0 0 0 1 0 0 0 0 0 0 0 0 0 0 0 0 0
  0 0 0 0 0 0 0 −1 0 0 0 0 0 0 0 −1 0 0 0 0 0 0 0 0 0 0 0 0 1 0 0 0 0 1 0 0 0 0 0 0 0 0 0 0 0
     0
  0 0 0 0 0 −1 0 1 0 0 0 0 0 −1 0 1 0 0 1 0 −1 0 0 0 0 0 0 0 −1 0 0 0 0 0 1 0 0 0 0 0 0 0 0 0
     0 0
  1 1 0 −1 0 1 0 −1 0 0 0 0 0 1 0 −1 0 0 0 0 1 0 0 0 0 0 0 0 1 0 0 0 0 0 0 1 0 0 0 0 0 0 0 0 0
      0
  0 0 0 0 0 0 0 0 0 0 0 0 0 0 0 0 0 0 −1 0 0 0 0 0 0 0 0 0 0 0 0 0 0 0 0 0 1 0 0 0 0 0 0 0 0 0
  0 0 0 0 0 −1 0 1 0 0 0 0 0 −1 0 1 0 0 1 0 −1 0 0 0 0 0 0 0 −1 0 0 0 0 0 0 0 0 1 0 0 0 0 0 0
     0 0
```

0 0 0 0 0 0 0 −1 0 0 0 0 0 0 0 −1 0 0 0 0 0 0 0 0 0 0 0 0 1 0 0 0 0 0 0 0 0 0 1 0 0 0 0 0 0
0
0 0 0 0 0 0 0 1 0 0 0 0 0 −1 0 1 0 0 0 0 −1 0 0 0 0 0 0 0 −1 0 0 0 0 0 0 0 0 0 0 1 0 0 0 0 0
0
1 0 −1 −1 0 1 0 −1 0 0 0 0 0 0 0 0 0 0 0 0 0 0 0 0 0 0 0 0 0 0 0 0 0 0 0 0 0 0 0 0 1 0 0 0 0
0
−1 0 1 1 0 0 0 1 0 0 0 0 0 1 0 0 0 0 −1 0 1 0 0 0 0 0 0 0 0 0 0 0 0 0 0 0 0 0 0 0 0 1 0 0 0 0
0 0 0 0 0 0 0 −1 0 0 0 0 0 0 0 0 0 0 0 0 0 0 0 0 0 0 0 0 0 0 0 0 0 0 0 0 0 0 0 0 0 0 1 0 0 0
1 0 0 0 0 −1 0 0 0 0 0 0 0 −1 0 0 0 0 1 0 −1 0 0 0 0 0 0 0 0 0 0 0 0 0 0 0 0 0 0 0 0 0 0 1 0
0
0 0 −1 −1 0 1 0 0 0 0 0 0 0 0 0 0 0 0 0 0 0 0 0 0 0 0 0 0 0 0 0 0 0 0 0 0 0 0 0 0 0 0 0 0 1
0
1 1 1 0 0 0 0 0 0 0 0 0 0 0 0 0 0 0 0 0 0 0 0 0 0 0 0 0 0 0 0 0 0 0 0 0 0 0 0 0 0 0 0 0 0 1

end

$$E_{13}+E_{14}-E_{34}\leq 1, \tag{67}$$
$$-E_{12}+E_{18}+E_{28}\leq 1, \tag{68}$$
$$E_{14}+E_{18}-E_{48}\leq 1, \tag{69}$$
$$E_{12}-E_{14}-E_{26}+E_{34}-E_{36}\leq -1, \tag{70}$$
$$E_{12}+E_{13}+E_{26}+E_{36}\leq 0, \tag{71}$$
$$-E_{13}-E_{14}+E_{16}-E_{18}+E_{36}+E_{48}\leq 0, \tag{72}$$
$$-E_{12}-E_{16}-E_{26}\leq 1, \tag{73}$$
$$E_{16}-E_{18}+E_{26}-E_{28}\leq 0, \tag{74}$$
$$E_{26}-E_{28}-E_{34}+E_{36}+E_{48}\leq 1, \tag{75}$$
$$E_{14}-E_{16}+E_{34}-E_{36}\leq 0, \tag{76}$$
$$-E_{13}-E_{14}-E_{26}+E_{28}-E_{36}-E_{48}\leq 0, \tag{77}$$
$$E_{12}-E_{14}-E_{15}\leq -1, \tag{78}$$
$$E_{13}+E_{14}-E_{16}-E_{17}\leq 0, \tag{79}$$
$$E_{12}-E_{14}+E_{16}-E_{18}-E_{19}\leq -1, \tag{80}$$
$$-E_{1,10}+E_{13}+E_{14}-E_{16}+E_{18}\leq 1, \tag{81}$$
$$-E_{12}-E_{13}-E_{23}\leq 1, \tag{82}$$
$$E_{12}-E_{14}-E_{24}\leq -1, \tag{83}$$
$$E_{14}-E_{25}\leq 0, \tag{84}$$
$$-E_{13}-E_{14}-E_{26}-E_{27}\leq 0, \tag{85}$$
$$E_{14}+E_{26}-E_{28}-E_{29}\leq 0, \tag{86}$$
$$-E_{12}-E_{13}-E_{14}-E_{2,10}-E_{26}+E_{28}\leq 0, \tag{87}$$
$$-E_{12}-E_{34}-E_{35}\leq 1, \tag{88}$$
$$E_{34}-E_{36}-E_{37}\leq -1, \tag{89}$$
$$E_{13}+E_{14}+E_{26}-E_{28}-E_{34}+E_{36}-E_{38}\leq 1, \tag{90}$$
$$-E_{12}-E_{13}-E_{14}-E_{26}+E_{28}-E_{39}\leq 0, \tag{91}$$
$$E_{14}+E_{26}-E_{28}-E_{3,10}\leq 0, \tag{92}$$
$$E_{12}-E_{45}\leq 0, \tag{93}$$
$$E_{34}-E_{36}-E_{46}\leq -1, \tag{94}$$
$$E_{36}-E_{47}\leq 0, \tag{95}$$
$$E_{12}+E_{34}-E_{36}-E_{48}-E_{49}\leq -1, \tag{96}$$
$$-E_{14}+E_{36}-E_{4,10}+E_{48}\leq 0, \tag{97}$$
$$E_{16}+E_{26}-E_{34}+E_{36}-E_{56}\leq 1, \tag{98}$$
$$-E_{16}-E_{26}-E_{36}-E_{57}\leq 0, \tag{99}$$
$$E_{18}+E_{28}-E_{48}-E_{58}\leq 0, \tag{100}$$
$$E_{16}-E_{18}+E_{26}-E_{28}-E_{34}+E_{36}+E_{48}-E_{59}\leq 0, \tag{101}$$
$$-E_{12}+E_{14}-E_{16}+E_{18}-E_{26}+E_{28}-E_{36}-E_{48}-E_{5,10}\leq 1, \tag{102}$$
$$E_{34}-E_{67}\leq 0, \tag{103}$$
$$E_{16}-E_{18}+E_{26}-E_{28}-E_{34}+E_{36}+E_{48}-E_{68}\leq 0, \tag{104}$$
$$E_{18}+E_{28}-E_{48}-E_{69}\leq 0, \tag{105}$$
$$-E_{18}+E_{26}-E_{28}+E_{36}+E_{48}-E_{6,10}\leq 0, \tag{106}$$
$$E_{13}+E_{14}-E_{16}+E_{18}-E_{78}\leq 1, \tag{107}$$
$$-E_{13}-E_{14}-E_{18}-E_{26}+E_{34}-E_{36}-E_{79}\leq -1, \tag{108}$$
$$E_{18}-E_{7,10}\leq 0, \tag{109}$$
$$E_{16}+E_{26}-E_{34}+E_{36}-E_{89}\leq 1, \tag{110}$$
$$E_{13}+E_{14}-E_{16}-E_{8,10}\leq 0, \tag{111}$$
$$-E_{12}-E_{13}-E_{9,10}\leq 1. \tag{112}$$

I. HOUSE/PENTAGON/PENTAGRAM GADGET

1. Bub-Stairs inequality

If one considers only the five probabilities on the intertwining atoms, then the following Bub-Stairs inequality $p_1 + p_3 + p_5 + p_7 + p_9 \leq 2$, among others, results:

```
* five probabilities on intertwining contexts
* p1, p3, p5, p7, p9
*
V-representation
begin
11  6  integer
1     1   0   0   0   0
1     1   0   1   0   0
1     1   0   0   1   0
1     0   1   0   0   0
1     0   1   0   1   0
1     0   1   0   0   1
1     0   0   1   0   0
1     0   0   1   0   1
1     0   0   0   1   0
1     0   0   0   0   1
1     0   0   0   0   0
end

~~~~~~ cddlib response

H-representation
begin
 11  6  real
  0  0  0  1  0  0
  1  0  0  0 -1 -1
  0  1  0  0  0  0
  1  0 -1 -1  0  0
  2 -1 -1 -1 -1 -1
  1 -1 -1  0  0  0
  0  0  0  0  1  0
  1 -1  0  0  0 -1
  1  0  0 -1 -1  0
  0  0  1  0  0  0
  0  0  0  0  0  1
end
```

One could also consider probabilities on the non-intertwining atoms yielding; in particular, $p_2 + p_4 + p_6 + p_8 + p_{10} \geq 1$.

```
* five probabilities
* on non-intertwining atoms
* p2, p4, p6, p8, p10
*
V-representation
begin
11  6  integer
1     0   1   1   1   0
1     0   0   0   1   0
1     0   1   0   0   0
1     0   0   1   1   1
1     0   0   0   0   1
1     0   0   1   0   0
1     1   0   0   1   1
1     1   0   0   0   0
1     1   1   0   0   1
```

```
1        1    1    1    0    0
1        1    1    1    1    1
end

~~~~~~ cddlib response

H-representation
begin
 11 6 real
  0  0  0  0  1  0
  0  0  0  0  0  1
  0  0  1  0  0  0
 -1  1  1  1  1  1
  0  1  0  0  0  0
  0  0  0  1  0  0
  1  1 -1  1 -1 -1
  1 -1  1 -1 -1  1
  1  1 -1 -1  1 -1
  1 -1  1 -1  1 -1
  1 -1 -1  1 -1  1
end
```

2. *Klyachko-Can-Biniciogolu-Shumovsky inequalities*

The following hull computation is limited to adjacent pair expectations; it yields the Klyachko-Can-Biniciogolu-Shumovsky inequality $E_{13}+E_{35}+E_{57}+E_{79}+E_{91}\geq 3$:

```
* five joint Expectations:
* E13 E35 E57 E79 E91
*
V-representation
begin
11  6   real
1       -1    1    1    1   -1
1       -1   -1   -1    1   -1
1       -1    1   -1   -1   -1
1       -1   -1    1    1    1
1       -1   -1   -1   -1    1
1       -1   -1    1   -1   -1
1        1   -1   -1    1    1
1        1   -1   -1   -1   -1
1        1    1   -1   -1    1
1        1    1    1   -1   -1
1        1    1    1    1    1
end

~~~~~~ cddlib response

H-representation
begin
 11 6 real
  1  0  0  0  1  0
  1  0  0  0  0  1
  1  0  1  0  0  0
  3  1  1  1  1  1
  1  1  0  0  0  0
  1  0  0  1  0  0
  1  1 -1  1 -1 -1
  1 -1  1 -1 -1  1
  1  1 -1 -1  1 -1
  1 -1  1 -1  1 -1
```

```
  1  −1  −1   1  −1   1
end
```

$$-E_{79} \leq 1 \quad (113)$$
$$-E_{91} \leq 1 \quad (114)$$
$$-E_{35} \leq 1 \quad (115)$$
$$-E_{13} - E_{35} - E_{57} - E_{79} - E_{91} \leq 3 \quad (116)$$
$$-E_{13} \leq 1 \quad (117)$$
$$-E_{57} \leq 1 \quad (118)$$
$$-E_{13} + E_{35} - E_{57} + E_{79} + E_{91} \leq 1 \quad (119)$$
$$+E_{13} - E_{35} + E_{57} + E_{79} - E_{91} \leq 1 \quad (120)$$
$$-E_{13} + E_{35} + E_{57} - E_{79} + E_{91} \leq 1 \quad (121)$$
$$+E_{13} - E_{35} + E_{57} - E_{79} + E_{91} \leq 1 \quad (122)$$
$$+E_{13} + E_{35} - E_{57} + E_{79} - E_{91} \leq 1. \quad (123)$$

A. Two intertwined pentagon logics forming a Specker Käfer (bug) or cat's cradle logic

1. *Probabilities on the Specker bug logic*

A *Mathematica* [3] code to reduce probabilities on the Specker bug logic:

```
Reduce[
p1 + p2 + p3 == 1
&& p3 + p4 + p5 == 1
&& p5 + p6 + p7 == 1
&&   p7 + p8 + p9 == 1
&& p9 + p10 + p11 == 1
&& p11 + p12 + p1 == 1
&&  p4 + p10 + p13 == 1,
{p3, p11, p5, p9, p4, p10}, Reals]

~~~~~~ Mathematica response

p1 == 3/2 - p12/2 - p13/2 - p2/2 - p6/2 - p7 - p8/2 &&
 p3 == -(1/2) + p12/2 + p13/2 - p2/2 + p6/2 + p7 + p8/2 &&
 p11 == -(1/2) - p12/2 + p13/2 + p2/2 + p6/2 + p7 + p8/2 &&
 p5 == 1 - p6 - p7 && p9 == 1 - p7 - p8 &&
 p4 == 1/2 - p12/2 - p13/2 + p2/2 + p6/2 - p8/2 &&
 p10 == 1/2 + p12/2 - p13/2 - p2/2 - p6/2 + p8/2
```

Computation of all the two-valued states thereon:

```
Reduce[p1 + p2 + p3 == 1 && p3 + p4 + p5 == 1 && p5 + p6 + p7 == 1 &&
  p7 + p8 + p9 == 1 && p9 + p10 + p11 == 1 && p11 + p12 + p1 == 1 &&
  p4 + p10 + p13 == 1 && p1^2 == p1 && p2^2 == p2 && p3^2 == p3 &&
  p4^2 == p4 && p5^2 == p5 && p6^2 == p6 && p7^2 == p7 && p8^2 == p8 &&
   p9^2 == p9 && p10^2 == p10 && p11^2 == p11 && p12^2 == p12 &&
  p13^2 == p13]

~~~~~~ Mathematica response

(p9 == 0 && p8 == 0 && p7 == 1 && p6 == 0 && p5 == 0 && p4 == 0 &&
   p3 == 1 && p2 == 0 && p13 == 0 && p12 == 1 && p11 == 0 &&
   p10 == 1 && p1 == 0) || (p9 == 0 && p8 == 0 && p7 == 1 && p6 == 0 &&
    p5 == 0 && p4 == 0 && p3 == 1 && p2 == 0 && p13 == 1 && p12 == 0 &&
```

```
  p11 == 1 && p10 == 0 && p1 == 0) || (p9 == 0 && p8 == 0 &&
 p7 == 1 && p6 == 0 && p5 == 0 && p4 == 1 && p3 == 0 && p2 == 1 &&
 p13 == 0 && p12 == 0 && p11 == 1 && p10 == 0 &&
 p1 == 0) || (p9 == 0 && p8 == 1 && p7 == 0 && p6 == 0 && p5 == 1 &&
  p4 == 0 && p3 == 0 && p2 == 0 && p13 == 0 && p12 == 0 &&
 p11 == 0 && p10 == 1 && p1 == 1) || (p9 == 0 && p8 == 1 &&
 p7 == 0 && p6 == 0 && p5 == 1 && p4 == 0 && p3 == 0 && p2 == 1 &&
 p13 == 0 && p12 == 1 && p11 == 0 && p10 == 1 &&
 p1 == 0) || (p9 == 0 && p8 == 1 && p7 == 0 && p6 == 0 && p5 == 1 &&
  p4 == 0 && p3 == 0 && p2 == 1 && p13 == 1 && p12 == 0 &&
 p11 == 1 && p10 == 0 && p1 == 0) || (p9 == 0 && p8 == 1 &&
 p7 == 0 && p6 == 1 && p5 == 0 && p4 == 0 && p3 == 1 && p2 == 0 &&
 p13 == 0 && p12 == 1 && p11 == 0 && p10 == 1 &&
 p1 == 0) || (p9 == 0 && p8 == 1 && p7 == 0 && p6 == 1 && p5 == 0 &&
  p4 == 0 && p3 == 1 && p2 == 0 && p13 == 1 && p12 == 0 &&
 p11 == 1 && p10 == 0 && p1 == 0) || (p9 == 0 && p8 == 1 &&
 p7 == 0 && p6 == 1 && p5 == 0 && p4 == 1 && p3 == 0 && p2 == 1 &&
 p13 == 0 && p12 == 0 && p11 == 1 && p10 == 0 &&
 p1 == 0) || (p9 == 1 && p8 == 0 && p7 == 0 && p6 == 0 && p5 == 1 &&
  p4 == 0 && p3 == 0 && p2 == 0 && p13 == 1 && p12 == 0 &&
 p11 == 0 && p10 == 0 && p1 == 1) || (p9 == 1 && p8 == 0 &&
 p7 == 0 && p6 == 0 && p5 == 1 && p4 == 0 && p3 == 0 && p2 == 1 &&
 p13 == 1 && p12 == 1 && p11 == 0 && p10 == 0 &&
 p1 == 0) || (p9 == 1 && p8 == 0 && p7 == 0 && p6 == 1 && p5 == 0 &&
  p4 == 0 && p3 == 1 && p2 == 0 && p13 == 1 && p12 == 1 &&
 p11 == 0 && p10 == 0 && p1 == 0) || (p9 == 1 && p8 == 0 &&
 p7 == 0 && p6 == 1 && p5 == 0 && p4 == 1 && p3 == 0 && p2 == 0 &&
 p13 == 0 && p12 == 0 && p11 == 0 && p10 == 0 &&
 p1 == 1) || (p9 == 1 && p8 == 0 && p7 == 0 && p6 == 1 && p5 == 0 &&
  p4 == 1 && p3 == 0 && p2 == 1 && p13 == 0 && p12 == 1 &&
 p11 == 0 && p10 == 0 && p1 == 0)
```

2. *Hull calculation for the probabilities on the Specker bug logic*

```
* 13 probabilities on atoms a1...a13:
* p1 ... p13
*
V-representation
begin
14 14 real
1 1 0 0 0 1 0 0 0 1 0 0 0 1
1 1 0 0 1 0 1 0 0 1 0 0 0 0
1 1 0 0 0 1 0 0 1 0 1 0 0 0
1 0 1 0 0 1 0 0 0 1 0 0 1 1
1 0 1 0 0 1 0 0 1 0 0 1 0 1
1 0 1 0 1 0 1 0 0 1 0 0 1 0
1 0 1 0 1 0 0 1 0 0 0 1 0 0
1 0 1 0 1 0 1 0 1 0 0 1 0 0
1 0 1 0 0 1 0 0 1 0 1 0 1 0
1 0 0 1 0 0 0 1 0 0 0 1 0 1
1 0 0 1 0 0 1 0 1 0 0 1 0 1
1 0 0 1 0 0 1 0 0 1 0 0 1 1
1 0 0 1 0 0 0 1 0 0 1 0 1 0
1 0 0 1 0 0 1 0 1 0 1 0 1 0
end

~~~~~~ cddlib response


H-representation
```

```
linearity 7  17 18 19 20 21 22 23
begin
 23 14 real
  0  0  0  0  1  0  0  0  0  0  0  0  0  0
  0  0  0  0  0  0  1  0  0  0  0  0  0  0
  0  1  1  0 -1  0  1  0 -1  0  0  0  0  0
  0  1  0  0  0  0  0  0  0  0  0  0  0  0
  0  1  1  0 -1  0  0  0  0  0  0  0  0  0
  0  1  2  0 -2  0  1  0 -1  0  0  0  0  0
  0  0  1  0 -1  0  1  0  0  0  0  0  0  0
  0  0  1  0  0  0  0  0  0  0  0  0  0  0
  0  0  0  0  0  0  0  0  0  0  1  0  0  0
  0  0  0  0  0  0  0  0  1  0  0  0  0  0
  0  0  1  0 -1  0  1  0 -1  0  1  0  0  0
  1  0  0  0 -1  0  0  0  0  0 -1  0  0  0
  1 -1 -1  0  1  0 -1  0  1  0 -1  0  0  0
  1 -1 -1  0  0  0  0  0  1  0 -1  0  0  0
  1 -1 -1  0  0  0  0  0  0  0  0  0  0  0
  1 -1 -1  0  1  0 -1  0  0  0  0  0  0  0
 -1  1  1  1  0  0  0  0  0  0  0  0  0  0
  0 -1 -1  0  1  1  0  0  0  0  0  0  0  0
 -1  1  1  0 -1  0  1  1  0  0  0  0  0  0
  0 -1 -1  0  1  0 -1  0  1  1  0  0  0  0
 -1  1  1  0 -1  0  1  0 -1  0  1  1  0  0
  0  0 -1  0  1  0 -1  0  1  0 -1  0  1  0
 -1  0  0  0  1  0  0  0  0  0  1  0  0  1
end
```

The resulting face inequalities are

$$-p_4 \leq 0, \tag{124}$$
$$-p_6 \leq 0, \tag{125}$$
$$-p_1 - p_2 + p_4 - p_6 + p_8 \leq 0, \tag{126}$$
$$-p_1 \leq 0, \tag{127}$$
$$-p_1 - p_2 + p_4 \leq 0, \tag{128}$$
$$-p_1 - 2p_2 + 2p_4 - p_6 + p_8 \leq 0, \tag{129}$$
$$-p_2 + p_4 - p_6 \leq 0, \tag{130}$$
$$-p_2 \leq 0, \tag{131}$$
$$-p_{10} \leq 0, \tag{132}$$
$$-p_8 \leq 0, \tag{133}$$
$$-p_2 + p_4 - p_6 + p_8 - p_{10} \leq 0, \tag{134}$$
$$+p_4 + p_{10} \leq +1, \tag{135}$$
$$+p_1 + p_2 - p_4 + p_6 - p_8 + p_{10} \leq +1, \tag{136}$$
$$+p_1 + p_2 - p_8 + p_{10} \leq +1, \tag{137}$$
$$+p_1 + p_2 \leq +1, \tag{138}$$
$$+p_1 + p_2 - p_4 + p_6 \leq +1, \tag{139}$$
$$-p_1 - p_2 - p_3 \leq -1, \tag{140}$$
$$+p_1 + p_2 - p_4 - p_5 \leq 0, \tag{141}$$
$$-p_1 - p_2 + p_4 - p_6 - p_7 \leq -1, \tag{142}$$
$$+p_1 + p_2 - p_4 + p_6 - p_8 - p_9 \leq 0, \tag{143}$$
$$-p_1 - p_2 + p_4 - p_6 + p_8 - p_{10} - p_{11} \leq -1, \tag{144}$$
$$+p_2 - p_4 + p_6 - p_8 + p_{10} - p_{12} \leq 0, \tag{145}$$
$$-p_4 - p_{10} - p_{13} \leq -1. \tag{146}$$

3. Hull calculation for the expectations on the Specker bug logic

```
* (13 expectations on atoms a1...a13:
* E1 ... E13 not enumerated)
* 6 joint expectations  E1*E3, E3*E5, ..., E11*E1
*
V-representation
begin
14  7  integer
1     -1   -1   -1   -1   -1   -1
1     -1    1    1   -1   -1   -1
1     -1   -1   -1    1    1   -1
1      1   -1   -1   -1   -1    1
1      1   -1   -1    1   -1   -1
1      1    1    1   -1   -1    1
1      1    1   -1   -1   -1   -1
1      1    1    1    1   -1   -1
1      1   -1   -1    1    1    1
1     -1   -1   -1   -1   -1   -1
1     -1   -1    1    1   -1   -1
1     -1   -1    1   -1   -1    1
1     -1   -1   -1   -1    1    1
1     -1   -1    1    1    1    1
end

~~~~~~ cddlib response


H-representation
linearity 1  18
begin
 18 7 real
  1  0  0  0  1  0  0
  1 -1  0  0  1 -1  0
  1 -1  1 -1  1 -1  0
  1  0  0 -1  1 -1  0
  1  0  1  0  0  0  0
  1  1  0  0  0  0  0
  1  1 -1  1  0  0  0
  1  0  0  1  0  0  0
  1  1 -1  0 -1  0  0
  1  0  0  0 -1  0  0
  1  0 -1  1 -1  0  0
  1  1 -1  1 -1  1  0
  1  0  0 -1  0  0  0
  1 -1  1 -1  0  0  0
  1 -1  0  0  0  0  0
  1  0  0  0  0  1  0
  0  0 -1  0  0 -1  0
  0 -1  1 -1  1 -1  1
end
```

4. Extended Specker bug logic

Here is the *Mathematica* [3] code to reduce probabilities on the extended (by two contexts) Specker bug logics:

```
Reduce[
p1 + p2 + p3 == 1
&& p3 + p4 + p5 == 1
```

```
&& p5 + p6 + p7 == 1
&&   p7 + p8 + p9 == 1
&& p9 + p10 + p11 == 1
&& p11 + p12 + p1 == 1
&&  p4 + p10 + p13 == 1
&& p1 + pc + q7 ==1
&& p7 + pc + q1 ==1,
{p3, p11, p5, p9, p4, p10, q3, q11, q5, q9, q4, q10, p13, q13, pc}]

~~~~~~ Mathematica response

p1 == p7 + q1 - q7 && p3 == 1 - p2 - p7 - q1 + q7 &&
 p11 == 1 - p12 - p7 - q1 + q7 && p5 == 1 - p6 - p7 &&
 p9 == 1 - p7 - p8 && p4 == -1 + p2 + p6 + 2 p7 + q1 - q7 &&
 p10 == -1 + p12 + 2 p7 + p8 + q1 - q7 &&
 p13 == 3 - p12 - p2 - p6 - 4 p7 - p8 - 2 q1 + 2 q7 &&
 pc == 1 - p7 - q1
```

Computation of all the 112 two-valued states thereon:

```
Reduce[p1 + p2 + p3 == 1 && p3 + p4 + p5 == 1 && p5 + p6 + p7 == 1 &&
  p7 + p8 + p9 == 1 && p9 + p10 + p11 == 1 && p11 + p12 + p1 == 1 &&
  p4 + p10 + p13 == 1 && p1^2 == p1 && p2^2 == p2 && p3^2 == p3 &&
  p4^2 == p4 && p5^2 == p5 && p6^2 == p6 && p7^2 == p7 && p8^2 == p8 &&
   p9^2 == p9 && p10^2 == p10 && p11^2 == p11 && p12^2 == p12 &&
  p13^2 == p13 &&  q1^2 == q1 &&  q7^2 == q7 &&  pc^2 == pc]

~~~~~~ Mathematica response

q7 == 0 && q1 == 0 && pc == 0 && p9 == 0 && p8 == 0 && p7 == 1 &&
   p6 == 0 && p5 == 0 && p4 == 0 && p3 == 1 && p2 == 0 && p13 == 0 &&
   p12 == 1 && p11 == 0 && p10 == 1 && p1 == 0) || (q7 == 0 &&
   q1 == 0 && pc == 0 && p9 == 0 && p8 == 0 && p7 == 1 && p6 == 0 &&
   p5 == 0 && p4 == 0 && p3 == 1 && p2 == 0 && p13 == 1 && p12 == 0 &&
    p11 == 1 && p10 == 0 && p1 == 0) ||
     [...]
  || (q7 == 1 && q1 == 1 && pc == 1 && p9 == 1 && p8 == 0 &&
    p7 == 0 && p6 == 1 && p5 == 0 && p4 == 1 && p3 == 0 && p2 == 1 &&
   p13 == 0 && p12 == 1 && p11 == 0 && p10 == 0 && p1 == 0)
```

B. Two intertwined Specker bug logics

Here is the *Mathematica* [3] code to reduce probabilities on two intertwined Specker bug logics:

```
Reduce[
p1 + p2 + p3 == 1
&& p3 + p4 + p5 == 1
&& p5 + p6 + p7 == 1
&&   p7 + p8 + p9 == 1
&& p9 + p10 + p11 == 1
&& p11 + p12 + p1 == 1
&&  p4 + p10 + p13 == 1
&& q1 + q2 + q3 == 1
&& q3 + q4 + q5 == 1
&& q5 + q6 + q7 == 1
&&   q7 + q8 + q9 == 1
&& q9 + q10 + q11 == 1
&& q11 + q12 + q1 == 1
&&  q4 + q10 + q13 == 1
&& p1 + pc + q7 ==1
```

```
&& p7 + pc + q1 ==1,
{p3, p11, p5, p9, p4, p10, q3, q11, q5, q9, q4, q10, p13, q13, pc}]

~~~~~~ Mathematica response

p1 == p7 + q1 - q7 && p3 == 1 - p2 - p7 - q1 + q7 &&
 p11 == 1 - p12 - p7 - q1 + q7 && p5 == 1 - p6 - p7 &&
 p9 == 1 - p7 - p8 && p4 == -1 + p2 + p6 + 2 p7 + q1 - q7 &&
 p10 == -1 + p12 + 2 p7 + p8 + q1 - q7 && q3 == 1 - q1 - q2 &&
 q11 == 1 - q1 - q12 && q5 == 1 - q6 - q7 && q9 == 1 - q7 - q8 &&
 q4 == -1 + q1 + q2 + q6 + q7 && q10 == -1 + q1 + q12 + q7 + q8 &&
 p13 == 3 - p12 - p2 - p6 - 4 p7 - p8 - 2 q1 + 2 q7 &&
 q13 == 3 - 2 q1 - q12 - q2 - q6 - 2 q7 - q8 && pc == 1 - p7 - q1
```

1. Hull calculation for the contexual inequalities corresponding to the Cabello, Estebaranz and García-Alcaine logic

```
* (13 expectations on atoms A1...A18:
*  not enumerated)
*  9 4th order expectations  A1A2A3A4 A4A5A6A7 ... A2A9A11A18
*
V-representation
begin
262144   10    real
1    1    1    1    1    1    1    1    1    1
1    1    1    1    1    1   -1   -1    1    1
1    1    1    1    1    1   -1    1    1   -1
[[...]]
1    1    1    1    1    1   -1    1    1   -1
1    1    1    1    1    1   -1   -1    1    1
1    1    1    1    1    1    1    1    1    1
end

~~~~~~ cddlib response

H-representation
begin
 274  10  real
  1  0  0  0  0  0  0  0  0  1
  1  0  0  0  0  0  0  0  1  0
  7 -1 -1 -1 -1 -1 -1  1  1  1
  7 -1 -1 -1 -1 -1  1 -1  1  1
  7 -1 -1 -1 -1  1 -1 -1  1  1
  7 -1 -1 -1  1 -1 -1 -1  1  1
  7 -1 -1  1 -1 -1 -1 -1  1  1
  7 -1  1 -1 -1 -1 -1 -1  1  1
  7  1 -1 -1 -1 -1 -1 -1  1  1
  1  0  0  0  0  0  0  1  0  0
  7 -1 -1 -1 -1 -1  1  1 -1  1
  7 -1 -1 -1 -1  1 -1  1 -1  1
  7 -1 -1 -1  1 -1 -1  1 -1  1
  7 -1 -1  1 -1 -1 -1  1 -1  1
  7 -1  1 -1 -1 -1 -1  1 -1  1
  7  1 -1 -1 -1 -1 -1  1 -1  1
  7 -1 -1 -1 -1 -1  1  1  1 -1
  7 -1 -1 -1 -1  1 -1  1  1 -1
  7 -1 -1 -1  1 -1 -1  1  1 -1
  7 -1 -1  1 -1 -1 -1  1  1 -1
  7 -1  1 -1 -1 -1 -1  1  1 -1
  7  1 -1 -1 -1 -1 -1  1  1 -1
  1  0  0  0  0  0  1  0  0  0
```

7	−1	−1	−1	−1	1	1	−1	−1	1
7	−1	−1	−1	1	−1	1	−1	−1	1
7	−1	−1	1	−1	−1	1	−1	−1	1
7	−1	1	−1	−1	−1	1	−1	−1	1
7	1	−1	−1	−1	−1	1	−1	−1	1
7	−1	−1	−1	−1	1	1	−1	1	−1
7	−1	−1	−1	1	−1	1	−1	1	−1
7	−1	−1	1	−1	−1	1	−1	1	−1
7	−1	1	−1	−1	−1	1	−1	1	−1
7	1	−1	−1	−1	−1	1	−1	1	−1
7	−1	−1	−1	−1	1	1	1	−1	−1
7	−1	−1	−1	1	−1	1	1	−1	−1
7	−1	−1	1	−1	−1	1	1	−1	−1
7	−1	1	−1	−1	−1	1	1	−1	−1
7	1	−1	−1	−1	−1	1	1	−1	−1
7	−1	−1	−1	−1	1	1	1	1	1
7	−1	−1	−1	1	−1	1	1	1	1
7	−1	−1	1	−1	−1	1	1	1	1
7	−1	1	−1	−1	−1	1	1	1	1
7	1	−1	−1	−1	−1	1	1	1	1
1	0	0	0	0	1	0	0	0	0
7	−1	−1	−1	1	1	−1	−1	−1	1
7	−1	−1	1	−1	1	−1	−1	−1	1
7	−1	1	−1	−1	1	−1	−1	−1	1
7	1	−1	−1	−1	1	−1	−1	−1	1
7	−1	−1	−1	1	1	−1	−1	1	−1
7	−1	−1	1	−1	1	−1	−1	1	−1
7	−1	1	−1	−1	1	−1	−1	1	−1
7	1	−1	−1	−1	1	−1	−1	1	−1
7	−1	−1	−1	1	1	−1	1	−1	−1
7	−1	−1	1	−1	1	−1	1	−1	−1
7	−1	1	−1	−1	1	−1	1	−1	−1
7	1	−1	−1	−1	1	−1	1	−1	−1
7	−1	−1	−1	1	1	−1	1	1	1
7	−1	−1	1	−1	1	−1	1	1	1
7	−1	1	−1	−1	1	−1	1	1	1
7	1	−1	−1	−1	1	−1	1	1	1
7	−1	−1	−1	1	1	1	−1	−1	−1
7	−1	−1	1	−1	1	1	−1	−1	−1
7	−1	1	−1	−1	1	1	−1	−1	−1
7	1	−1	−1	−1	1	1	−1	−1	−1
7	−1	−1	−1	1	1	1	−1	1	1
7	−1	−1	1	−1	1	1	−1	1	1
7	−1	1	−1	−1	1	1	−1	1	1
7	1	−1	−1	−1	1	1	−1	1	1
7	−1	−1	−1	1	1	1	1	−1	1
7	−1	−1	1	−1	1	1	1	−1	1
7	−1	1	−1	−1	1	1	1	−1	1
7	1	−1	−1	−1	1	1	1	−1	1
7	−1	−1	−1	1	1	1	1	1	−1
7	−1	−1	1	−1	1	1	1	1	−1
7	−1	1	−1	−1	1	1	1	1	−1
7	1	−1	−1	−1	1	1	1	1	−1
1	0	0	0	1	0	0	0	0	0
7	−1	−1	1	1	−1	−1	−1	−1	1
7	−1	1	−1	1	−1	−1	−1	−1	1
7	1	−1	−1	1	−1	−1	−1	−1	1
7	−1	−1	1	1	−1	−1	−1	1	−1
7	−1	1	−1	1	−1	−1	−1	1	−1
7	1	−1	−1	1	−1	−1	−1	1	−1
7	−1	−1	1	1	−1	−1	1	−1	−1
7	−1	1	−1	1	−1	−1	1	−1	−1
7	1	−1	−1	1	−1	−1	1	−1	−1
7	−1	−1	1	1	−1	−1	1	1	1

7	−1	1	−1	1	−1	−1	1	1	1
7	1	−1	−1	1	−1	−1	1	1	1
7	−1	−1	1	1	−1	1	−1	−1	−1
7	−1	1	−1	1	−1	1	−1	−1	−1
7	1	−1	−1	1	−1	1	−1	−1	−1
7	−1	−1	1	1	−1	1	−1	1	1
7	−1	1	−1	1	−1	1	−1	1	1
7	1	−1	−1	1	−1	1	−1	1	1
7	−1	−1	1	1	−1	1	1	−1	1
7	−1	1	−1	1	−1	1	1	−1	1
7	1	−1	−1	1	−1	1	1	−1	1
7	−1	−1	1	1	−1	1	1	1	−1
7	−1	1	−1	1	−1	1	1	1	−1
7	1	−1	−1	1	−1	1	1	1	−1
7	−1	−1	1	1	1	−1	−1	−1	−1
7	−1	1	−1	1	1	−1	−1	−1	−1
7	1	−1	−1	1	1	−1	−1	−1	−1
7	−1	−1	1	1	1	−1	−1	1	1
7	−1	1	−1	1	1	−1	−1	1	1
7	1	−1	−1	1	1	−1	−1	1	1
7	−1	−1	1	1	1	−1	1	−1	1
7	−1	1	−1	1	1	−1	1	−1	1
7	1	−1	−1	1	1	−1	1	−1	1
7	−1	−1	1	1	1	−1	1	1	−1
7	−1	1	−1	1	1	−1	1	1	−1
7	1	−1	−1	1	1	−1	1	1	−1
7	−1	−1	1	1	1	1	−1	−1	1
7	−1	1	−1	1	1	1	−1	−1	1
7	1	−1	−1	1	1	1	−1	−1	1
7	−1	−1	1	1	1	1	−1	1	−1
7	−1	1	−1	1	1	1	−1	1	−1
7	1	−1	−1	1	1	1	−1	1	−1
7	−1	−1	1	1	1	1	1	−1	−1
7	−1	1	−1	1	1	1	1	−1	−1
7	1	−1	−1	1	1	1	1	−1	−1
7	−1	−1	1	1	1	1	1	1	1
7	−1	1	−1	1	1	1	1	1	1
7	1	−1	−1	1	1	1	1	1	1
1	0	0	1	0	0	0	0	0	0
7	−1	1	1	−1	−1	−1	−1	−1	1
7	1	−1	1	−1	−1	−1	−1	−1	1
7	−1	1	1	−1	−1	−1	−1	1	−1
7	1	−1	1	−1	−1	−1	−1	1	−1
7	−1	1	1	−1	−1	−1	1	−1	−1
7	1	−1	1	−1	−1	−1	1	−1	−1
7	−1	1	1	−1	−1	−1	1	1	1
7	1	−1	1	−1	−1	−1	1	1	1
7	−1	1	1	−1	−1	1	−1	−1	−1
7	1	−1	1	−1	−1	1	−1	−1	−1
7	−1	1	1	−1	−1	1	−1	1	1
7	1	−1	1	−1	−1	1	−1	1	1
7	−1	1	1	−1	−1	1	1	−1	1
7	1	−1	1	−1	−1	1	1	−1	1
7	−1	1	1	−1	−1	1	1	1	−1
7	1	−1	1	−1	−1	1	1	1	−1
7	−1	1	1	−1	1	−1	−1	−1	−1
7	1	−1	1	−1	1	−1	−1	−1	−1
7	−1	1	1	−1	1	−1	−1	1	1
7	1	−1	1	−1	1	−1	−1	1	1
7	−1	1	1	−1	1	−1	1	−1	1
7	1	−1	1	−1	1	−1	1	−1	1
7	−1	1	1	−1	1	−1	1	1	−1
7	1	−1	1	−1	1	−1	1	1	−1
7	−1	1	1	−1	1	1	−1	−1	1

7	1	−1	1	−1	1	1	−1	−1	1
7	−1	1	1	−1	1	1	−1	1	−1
7	1	−1	1	−1	1	1	−1	1	−1
7	−1	1	1	−1	1	1	1	−1	−1
7	1	−1	1	−1	1	1	1	−1	−1
7	−1	1	1	−1	1	1	1	1	1
7	1	−1	1	−1	1	1	1	1	1
7	−1	1	1	1	−1	−1	−1	−1	−1
7	1	−1	1	1	−1	−1	−1	−1	−1
7	−1	1	1	1	−1	−1	−1	1	1
7	1	−1	1	1	−1	−1	−1	1	1
7	−1	1	1	1	−1	−1	1	−1	1
7	1	−1	1	1	−1	−1	1	−1	1
7	−1	1	1	1	−1	−1	1	1	−1
7	1	−1	1	1	−1	−1	1	1	−1
7	−1	1	1	1	−1	1	−1	−1	1
7	1	−1	1	1	−1	1	−1	−1	1
7	−1	1	1	1	−1	1	−1	1	−1
7	1	−1	1	1	−1	1	−1	1	−1
7	−1	1	1	1	−1	1	1	−1	−1
7	1	−1	1	1	−1	1	1	−1	−1
7	−1	1	1	1	−1	1	1	1	1
7	1	−1	1	1	−1	1	1	1	1
7	−1	1	1	1	1	−1	−1	−1	1
7	1	−1	1	1	1	−1	−1	−1	1
7	−1	1	1	1	1	−1	−1	1	−1
7	1	−1	1	1	1	−1	−1	1	−1
7	−1	1	1	1	1	−1	1	−1	−1
7	1	−1	1	1	1	−1	1	−1	−1
7	−1	1	1	1	1	−1	1	1	1
7	1	−1	1	1	1	−1	1	1	1
7	−1	1	1	1	1	1	−1	−1	−1
7	1	−1	1	1	1	1	−1	−1	−1
7	−1	1	1	1	1	1	−1	1	1
7	1	−1	1	1	1	1	−1	1	1
7	−1	1	1	1	1	1	1	−1	1
7	1	−1	1	1	1	1	1	−1	1
7	−1	1	1	1	1	1	1	1	−1
7	1	−1	1	1	1	1	1	1	−1
1	0	1	0	0	0	0	0	0	0
7	1	1	−1	−1	−1	−1	−1	−1	1
7	1	1	−1	−1	−1	−1	−1	1	−1
7	1	1	−1	−1	−1	−1	1	−1	−1
7	1	1	−1	−1	−1	−1	1	1	1
7	1	1	−1	−1	−1	1	−1	−1	−1
7	1	1	−1	−1	−1	1	−1	1	1
7	1	1	−1	−1	−1	1	1	−1	1
7	1	1	−1	−1	−1	1	1	1	−1
7	1	1	−1	−1	1	−1	−1	−1	−1
7	1	1	−1	−1	1	−1	−1	1	1
7	1	1	−1	−1	1	−1	1	−1	1
7	1	1	−1	−1	1	−1	1	1	−1
7	1	1	−1	−1	1	1	−1	−1	1
7	1	1	−1	−1	1	1	−1	1	−1
7	1	1	−1	−1	1	1	1	−1	−1
7	1	1	−1	−1	1	1	1	1	1
7	1	1	−1	1	−1	−1	−1	−1	−1
7	1	1	−1	1	−1	−1	−1	1	1
7	1	1	−1	1	−1	−1	1	−1	1
7	1	1	−1	1	−1	−1	1	1	−1
7	1	1	−1	1	−1	1	−1	−1	1
7	1	1	−1	1	−1	1	−1	1	−1
7	1	1	−1	1	−1	1	1	−1	−1
7	1	1	−1	1	−1	1	1	1	1

```
  7  1  1 -1  1  1 -1 -1 -1  1
  7  1  1 -1  1  1 -1 -1  1 -1
  7  1  1 -1  1  1 -1  1 -1 -1
  7  1  1 -1  1  1 -1  1  1  1
  7  1  1 -1  1  1  1 -1 -1 -1
  7  1  1 -1  1  1  1 -1  1  1
  7  1  1 -1  1  1  1  1 -1  1
  7  1  1 -1  1  1  1  1  1 -1
  7  1  1  1 -1 -1 -1 -1 -1 -1
  7  1  1  1 -1 -1 -1 -1  1  1
  7  1  1  1 -1 -1 -1  1 -1  1
  7  1  1  1 -1 -1 -1  1  1 -1
  7  1  1  1 -1 -1  1 -1 -1  1
  7  1  1  1 -1 -1  1 -1  1 -1
  7  1  1  1 -1 -1  1  1 -1 -1
  7  1  1  1 -1 -1  1  1  1  1
  7  1  1  1 -1  1 -1 -1 -1  1
  7  1  1  1 -1  1 -1 -1  1 -1
  7  1  1  1 -1  1 -1  1 -1 -1
  7  1  1  1 -1  1 -1  1  1  1
  7  1  1  1 -1  1  1 -1 -1 -1
  7  1  1  1 -1  1  1 -1  1  1
  7  1  1  1 -1  1  1  1 -1  1
  7  1  1  1 -1  1  1  1  1 -1
  7  1  1  1  1 -1 -1 -1 -1  1
  7  1  1  1  1 -1 -1 -1  1 -1
  7  1  1  1  1 -1 -1  1 -1 -1
  7  1  1  1  1 -1 -1  1  1  1
  7  1  1  1  1 -1  1 -1 -1 -1
  7  1  1  1  1 -1  1 -1  1  1
  7  1  1  1  1 -1  1  1 -1  1
  7  1  1  1  1 -1  1  1  1 -1
  7  1  1  1  1  1 -1 -1 -1 -1
  7  1  1  1  1  1 -1 -1  1  1
  7  1  1  1  1  1 -1  1 -1  1
  7  1  1  1  1  1 -1  1  1 -1
  7  1  1  1  1  1  1 -1 -1  1
  7  1  1  1  1  1  1 -1  1 -1
  7  1  1  1  1  1  1  1 -1 -1
  7  1  1  1  1  1  1  1  1  1
  1  1  0  0  0  0  0  0  0  0
  7  1 -1 -1 -1 -1 -1 -1 -1 -1
  7 -1  1 -1 -1 -1 -1 -1 -1 -1
  7 -1 -1  1 -1 -1 -1 -1 -1 -1
  7 -1 -1 -1  1 -1 -1 -1 -1 -1
  7 -1 -1 -1 -1  1 -1 -1 -1 -1
  7 -1 -1 -1 -1 -1  1 -1 -1 -1
  7 -1 -1 -1 -1 -1 -1  1 -1 -1
  7 -1 -1 -1 -1 -1 -1 -1  1 -1
  7 -1 -1 -1 -1 -1 -1 -1 -1  1
  1  0  0  0  0  0  0  0  0 -1
  1  0  0  0  0  0  0  0 -1  0
  1  0  0  0  0  0  0 -1  0  0
  1  0  0  0  0  0 -1  0  0  0
  1  0  0  0  0 -1  0  0  0  0
  1  0  0  0 -1  0  0  0  0  0
  1  0  0 -1  0  0  0  0  0  0
  1  0 -1  0  0  0  0  0  0  0
  1 -1  0  0  0  0  0  0  0  0
end

~~~~~~ cddlib reverse vertex computation
```

```
V-representation
begin
 256  10  real
  1 -1 -1 -1 -1 -1 -1  1  1  1
  1 -1 -1 -1 -1 -1  1 -1  1  1
  1 -1 -1 -1 -1  1 -1 -1  1  1
  1 -1 -1 -1  1 -1 -1 -1  1  1
  1 -1 -1  1 -1 -1 -1 -1  1  1
  1 -1  1 -1 -1 -1 -1 -1  1  1
  1  1 -1 -1 -1 -1 -1 -1  1  1
  1  1 -1 -1 -1 -1 -1  1  1 -1
  1 -1  1 -1 -1 -1 -1  1  1 -1
  1 -1 -1  1 -1 -1 -1  1  1 -1
  1 -1 -1 -1  1 -1 -1  1  1 -1
  1 -1 -1 -1 -1  1 -1  1  1 -1
  1 -1 -1 -1 -1 -1  1  1  1 -1
  1  1 -1 -1 -1 -1  1 -1  1 -1
  1 -1  1 -1 -1 -1  1 -1  1 -1
  1 -1 -1  1 -1 -1  1 -1  1 -1
  1 -1 -1 -1  1 -1  1 -1  1 -1
  1 -1 -1 -1 -1  1  1 -1  1 -1
  1  1 -1 -1 -1 -1  1  1  1  1
  1 -1  1 -1 -1 -1  1  1  1  1
  1 -1 -1  1 -1 -1  1  1  1  1
  1 -1 -1 -1  1 -1  1  1  1  1
  1 -1 -1 -1 -1  1  1  1  1  1
  1  1 -1 -1 -1  1 -1 -1  1 -1
  1 -1  1 -1 -1  1 -1 -1  1 -1
  1 -1 -1  1 -1  1 -1 -1  1 -1
  1 -1 -1 -1  1  1 -1 -1  1 -1
  1  1 -1 -1 -1  1 -1  1  1  1
  1 -1  1 -1 -1  1 -1  1  1  1
  1 -1 -1  1 -1  1 -1  1  1  1
  1 -1 -1 -1  1  1 -1  1  1  1
  1  1 -1 -1 -1  1  1 -1  1  1
  1 -1  1 -1 -1  1  1 -1  1  1
  1 -1 -1  1 -1  1  1 -1  1  1
  1 -1 -1 -1  1  1  1 -1  1  1
  1  1 -1 -1 -1  1  1  1  1 -1
  1 -1  1 -1 -1  1  1  1  1 -1
  1 -1 -1  1 -1  1  1  1  1 -1
  1 -1 -1 -1  1  1  1  1  1 -1
  1  1 -1 -1  1 -1 -1 -1  1 -1
  1 -1  1 -1  1 -1 -1 -1  1 -1
  1 -1 -1  1  1 -1 -1 -1  1 -1
  1  1 -1 -1  1 -1 -1  1  1  1
  1 -1  1 -1  1 -1 -1  1  1  1
  1 -1 -1  1  1 -1 -1  1  1  1
  1  1 -1 -1  1 -1  1 -1  1  1
  1 -1  1 -1  1 -1  1 -1  1  1
  1 -1 -1  1  1 -1  1 -1  1  1
  1  1 -1 -1  1 -1  1  1  1 -1
  1 -1  1 -1  1 -1  1  1  1 -1
  1 -1 -1  1  1 -1  1  1  1 -1
  1  1 -1 -1  1  1 -1 -1  1  1
  1 -1  1 -1  1  1 -1 -1  1  1
  1 -1 -1  1  1  1 -1 -1  1  1
  1  1 -1 -1  1  1 -1  1  1 -1
  1 -1  1 -1  1  1 -1  1  1 -1
  1 -1 -1  1  1  1 -1  1  1 -1
  1  1 -1 -1  1  1  1 -1  1 -1
  1 -1  1 -1  1  1  1 -1  1 -1
  1 -1 -1  1  1  1  1 -1  1 -1
  1  1 -1 -1  1  1  1  1  1  1
```

1	−1	1	−1	1	1	1	1	1	1
1	−1	−1	1	1	1	1	1	1	1
1	1	−1	1	−1	−1	−1	−1	1	−1
1	−1	1	1	−1	−1	−1	−1	1	−1
1	1	−1	1	−1	−1	−1	1	1	1
1	−1	1	1	−1	−1	−1	1	1	1
1	1	−1	1	−1	−1	1	−1	1	1
1	−1	1	1	−1	−1	1	−1	1	1
1	1	−1	1	−1	−1	1	1	1	−1
1	−1	1	1	−1	−1	1	1	1	−1
1	1	−1	1	−1	1	−1	−1	1	1
1	−1	1	1	−1	1	−1	−1	1	1
1	1	−1	1	−1	1	−1	1	1	−1
1	−1	1	1	−1	1	−1	1	1	−1
1	1	−1	1	−1	1	1	−1	1	−1
1	−1	1	1	−1	1	1	−1	1	−1
1	1	−1	1	−1	1	1	1	1	1
1	−1	1	1	−1	1	1	1	1	1
1	1	−1	1	1	−1	−1	−1	1	1
1	−1	1	1	1	−1	−1	−1	1	1
1	1	−1	1	1	−1	−1	1	1	−1
1	−1	1	1	1	−1	−1	1	1	−1
1	1	−1	1	1	−1	1	−1	1	−1
1	−1	1	1	1	−1	1	−1	1	−1
1	1	−1	1	1	−1	1	1	1	1
1	−1	1	1	1	−1	1	1	1	1
1	1	−1	1	1	1	−1	−1	1	−1
1	−1	1	1	1	1	−1	−1	1	−1
1	1	−1	1	1	1	−1	1	1	1
1	−1	1	1	1	1	−1	1	1	1
1	1	−1	1	1	1	1	−1	1	1
1	−1	1	1	1	1	1	−1	1	1
1	1	−1	1	1	1	1	1	1	−1
1	−1	1	1	1	1	1	1	1	−1
1	1	1	−1	−1	−1	−1	−1	1	−1
1	1	1	−1	−1	−1	−1	1	1	1
1	1	1	−1	−1	−1	1	−1	1	1
1	1	1	−1	−1	−1	1	1	1	−1
1	1	1	−1	−1	1	−1	−1	1	1
1	1	1	−1	−1	1	−1	1	1	−1
1	1	1	−1	−1	1	1	−1	1	−1
1	1	1	−1	−1	1	1	1	1	1
1	1	1	−1	1	−1	−1	−1	1	1
1	1	1	−1	1	−1	−1	1	1	−1
1	1	1	−1	1	−1	1	−1	1	−1
1	1	1	−1	1	−1	1	1	1	1
1	1	1	−1	1	1	−1	−1	1	−1
1	1	1	−1	1	1	−1	1	1	1
1	1	1	−1	1	1	1	−1	1	1
1	1	1	−1	1	1	1	1	1	−1
1	1	1	1	−1	−1	−1	−1	1	1
1	1	1	1	−1	−1	−1	1	1	−1
1	1	1	1	−1	−1	1	−1	1	−1
1	1	1	1	−1	−1	1	1	1	1
1	1	1	1	−1	1	−1	−1	1	−1
1	1	1	1	−1	1	−1	1	1	1
1	1	1	1	−1	1	1	−1	1	1
1	1	1	1	−1	1	1	1	1	−1
1	1	1	1	1	−1	−1	−1	1	−1
1	1	1	1	1	−1	−1	1	1	1
1	1	1	1	1	−1	1	−1	1	1
1	1	1	1	1	−1	1	1	1	−1
1	1	1	1	1	1	−1	−1	1	1
1	1	1	1	1	1	−1	1	1	−1

1	1	1	1	1	1	1	−1	1	−1
1	1	1	1	1	1	1	1	1	1
1	1	1	1	1	1	1	1	−1	−1
1	1	1	1	1	1	1	−1	−1	1
1	1	1	1	1	1	−1	1	−1	1
1	1	1	1	1	1	−1	−1	−1	−1
1	1	1	1	1	−1	1	1	−1	1
1	1	1	1	1	−1	1	−1	−1	−1
1	1	1	1	1	−1	−1	1	−1	−1
1	1	1	1	1	−1	−1	−1	−1	1
1	1	1	1	−1	1	1	1	−1	1
1	1	1	1	−1	1	1	−1	−1	−1
1	1	1	1	−1	1	−1	1	−1	−1
1	1	1	1	−1	1	−1	−1	−1	1
1	1	1	1	−1	−1	1	1	−1	−1
1	1	1	1	−1	−1	1	−1	−1	1
1	1	1	1	−1	−1	−1	1	−1	1
1	1	1	1	−1	−1	−1	−1	−1	−1
1	1	1	−1	1	1	1	1	−1	1
1	1	1	−1	1	1	1	−1	−1	−1
1	1	1	−1	1	1	−1	1	−1	−1
1	1	1	−1	1	1	−1	−1	−1	1
1	1	1	−1	1	−1	1	1	−1	−1
1	1	1	−1	1	−1	1	−1	−1	1
1	1	1	−1	1	−1	−1	1	−1	1
1	1	1	−1	1	−1	−1	−1	−1	−1
1	1	1	−1	−1	1	1	1	−1	−1
1	1	1	−1	−1	1	1	−1	−1	1
1	1	1	−1	−1	1	−1	1	−1	1
1	1	1	−1	−1	1	−1	−1	−1	−1
1	1	1	−1	−1	−1	1	1	−1	1
1	1	1	−1	−1	−1	1	−1	−1	−1
1	1	1	−1	−1	−1	−1	1	−1	−1
1	1	1	−1	−1	−1	−1	−1	−1	1
1	−1	1	1	1	1	1	1	−1	1
1	1	−1	1	1	1	1	1	−1	1
1	−1	1	1	1	1	1	−1	−1	−1
1	1	−1	1	1	1	1	−1	−1	−1
1	−1	1	1	1	1	−1	1	−1	−1
1	1	−1	1	1	1	−1	1	−1	−1
1	−1	1	1	1	1	−1	−1	−1	1
1	1	−1	1	1	1	−1	−1	−1	1
1	−1	1	1	1	−1	1	1	−1	−1
1	1	−1	1	1	−1	1	1	−1	−1
1	−1	1	1	1	−1	1	−1	−1	1
1	1	−1	1	1	−1	1	−1	−1	1
1	−1	1	1	1	−1	−1	1	−1	1
1	1	−1	1	1	−1	−1	1	−1	1
1	−1	1	1	1	−1	−1	−1	−1	−1
1	1	−1	1	1	−1	−1	−1	−1	−1
1	−1	1	1	−1	1	1	1	−1	−1
1	1	−1	1	−1	1	1	1	−1	−1
1	−1	1	1	−1	1	1	−1	−1	1
1	1	−1	1	−1	1	1	−1	−1	1
1	−1	1	1	−1	1	−1	1	−1	1
1	1	−1	1	−1	1	−1	1	−1	1
1	−1	1	1	−1	1	−1	−1	−1	−1
1	1	−1	1	−1	1	−1	−1	−1	−1
1	−1	1	1	−1	−1	1	1	−1	1
1	1	−1	1	−1	−1	1	1	−1	1
1	−1	1	1	−1	−1	1	−1	−1	−1
1	1	−1	1	−1	−1	1	−1	−1	−1
1	−1	1	1	−1	−1	−1	1	−1	−1
1	1	−1	1	−1	−1	−1	1	−1	−1

1	−1	1	1	−1	−1	−1	−1	−1	1
1	1	−1	1	−1	−1	−1	−1	−1	1
1	−1	−1	1	1	1	1	1	−1	−1
1	−1	1	−1	1	1	1	1	−1	−1
1	1	−1	−1	1	1	1	1	−1	−1
1	−1	−1	1	1	1	1	−1	−1	1
1	−1	1	−1	1	1	1	−1	−1	1
1	1	−1	−1	1	1	1	−1	−1	1
1	−1	−1	1	1	1	−1	1	−1	1
1	−1	1	−1	1	1	−1	1	−1	1
1	1	−1	−1	1	1	−1	1	−1	1
1	−1	−1	1	1	1	−1	−1	−1	−1
1	−1	1	−1	1	1	−1	−1	−1	−1
1	1	−1	−1	1	1	−1	−1	−1	−1
1	−1	−1	1	1	−1	1	1	−1	1
1	−1	1	−1	1	−1	1	1	−1	1
1	1	−1	−1	1	−1	1	1	−1	1
1	−1	−1	1	1	−1	1	−1	−1	−1
1	−1	1	−1	1	−1	1	−1	−1	−1
1	1	−1	−1	1	−1	1	−1	−1	−1
1	−1	−1	1	1	−1	−1	1	−1	−1
1	−1	1	−1	1	−1	−1	1	−1	−1
1	1	−1	−1	1	−1	−1	1	−1	−1
1	−1	−1	1	1	−1	−1	−1	−1	1
1	−1	1	−1	1	−1	−1	−1	−1	1
1	1	−1	−1	1	−1	−1	−1	−1	1
1	−1	−1	−1	1	1	1	1	−1	1
1	−1	−1	1	−1	1	1	1	−1	1
1	−1	1	−1	−1	1	1	1	−1	1
1	1	−1	−1	−1	1	1	1	−1	1
1	−1	−1	−1	1	1	1	−1	−1	−1
1	−1	−1	1	−1	1	1	−1	−1	−1
1	−1	1	−1	−1	1	1	−1	−1	−1
1	1	−1	−1	−1	1	1	−1	−1	−1
1	−1	−1	−1	1	1	−1	1	−1	−1
1	−1	−1	1	−1	1	−1	1	−1	−1
1	−1	1	−1	−1	1	−1	1	−1	−1
1	1	−1	−1	−1	1	−1	1	−1	−1
1	−1	−1	−1	1	1	−1	−1	−1	1
1	−1	−1	1	−1	1	−1	−1	−1	1
1	−1	1	−1	−1	1	−1	−1	−1	1
1	1	−1	−1	−1	1	−1	−1	−1	1
1	−1	−1	−1	−1	1	1	1	−1	−1
1	−1	−1	−1	1	−1	1	1	−1	−1
1	−1	−1	1	−1	−1	1	1	−1	−1
1	−1	1	−1	−1	−1	1	1	−1	−1
1	1	−1	−1	−1	−1	1	1	−1	−1
1	−1	−1	−1	−1	1	1	−1	−1	1
1	−1	−1	−1	1	−1	1	−1	−1	1
1	−1	−1	1	−1	−1	1	−1	−1	1
1	−1	1	−1	−1	−1	1	−1	−1	1
1	1	−1	−1	−1	−1	1	−1	−1	1
1	−1	−1	−1	−1	−1	1	1	−1	1
1	−1	−1	−1	−1	1	−1	1	−1	1
1	−1	−1	−1	1	−1	−1	1	−1	1
1	−1	−1	1	−1	−1	−1	1	−1	1
1	−1	1	−1	−1	−1	−1	1	−1	1
1	1	−1	−1	−1	−1	−1	1	−1	1
1	−1	−1	−1	−1	−1	−1	1	−1	−1
1	−1	−1	−1	−1	−1	1	−1	−1	−1
1	−1	−1	−1	−1	1	−1	−1	−1	−1
1	−1	−1	−1	1	−1	−1	−1	−1	−1
1	−1	−1	1	−1	−1	−1	−1	−1	−1
1	−1	1	−1	−1	−1	−1	−1	−1	−1

```
  1   1 −1 −1 −1 −1 −1 −1 −1 −1
  1 −1 −1 −1 −1 −1 −1 −1   1 −1
  1 −1 −1 −1 −1 −1 −1 −1 −1   1
end
```

2. Hull calculation for the contexual inequalities corresponding to the pentagon logic

```
* (10 expectations on atoms A1...A10:
*  not enumerated)
*  5 3th order expectations  A1A2A3 A3A4A5 ... A9A10A1
*  obtained through reverse Hull computation
V−representation
begin
 32 6 real
  1   1 −1 −1 −1 −1
  1   1 −1 −1 −1   1
  1   1 −1 −1   1 −1
  1   1 −1 −1   1   1
  1   1 −1   1 −1 −1
  1   1 −1   1 −1   1
  1   1 −1   1   1 −1
  1   1 −1   1   1   1
  1   1   1   1 −1 −1
  1   1   1   1 −1   1
  1   1   1   1   1   1
  1   1   1   1   1 −1
  1   1   1 −1   1   1
  1   1   1 −1   1 −1
  1   1   1 −1 −1   1
  1   1   1 −1 −1 −1
  1 −1   1   1   1   1
  1 −1   1   1   1 −1
  1 −1   1   1 −1   1
  1 −1   1   1 −1 −1
  1 −1   1 −1   1   1
  1 −1   1 −1   1 −1
  1 −1   1 −1 −1   1
  1 −1   1 −1 −1 −1
  1 −1 −1   1   1   1
  1 −1 −1   1   1 −1
  1 −1 −1   1 −1   1
  1 −1 −1   1 −1 −1
  1 −1 −1 −1   1   1
  1 −1 −1 −1   1 −1
  1 −1 −1 −1 −1   1
  1 −1 −1 −1 −1 −1
end

~~~~~~ cddlib response

H−representation
begin
 10 6 real
  1   0   0   0   0   1
  1   0   0   0   1   0
  1   0   0   1   0   0
  1   0   1   0   0   0
  1   1   0   0   0   0
  1   0   0   0   0 −1
  1   0   0   0 −1   0
```

```
  1   0   0  -1   0   0
  1   0  -1   0   0   0
  1  -1   0   0   0   0
end
```

3. Hull calculation for the contexual inequalities corresponding to Specker bug logics

```
* (13 expectations on atoms A1...A13:
*  not enumerated)
*  7 3th order expectations  A1A2A3 A3A4A5 ... A11A12A1 A4A13A10
*  obtained through reverse Hull computation
V-representation
begin
 128 8 real
  1   1  -1  -1  -1  -1  -1  -1
  1   1  -1  -1  -1  -1  -1   1
  1   1  -1  -1  -1  -1   1  -1
  1   1  -1  -1  -1  -1   1   1
  1   1  -1  -1  -1   1  -1  -1
  1   1  -1  -1  -1   1  -1   1
  1   1  -1  -1  -1   1   1  -1
  1   1  -1  -1  -1   1   1   1
  1   1  -1  -1   1  -1  -1  -1
  1   1  -1  -1   1  -1  -1   1
  1   1  -1  -1   1  -1   1  -1
  1   1  -1  -1   1  -1   1   1
  1   1  -1  -1   1   1  -1  -1
  1   1  -1  -1   1   1  -1   1
  1   1  -1  -1   1   1   1  -1
  1   1  -1  -1   1   1   1   1
  1   1  -1   1  -1  -1  -1  -1
  1   1  -1   1  -1  -1  -1   1
  1   1  -1   1  -1  -1   1  -1
  1   1  -1   1  -1  -1   1   1
  1   1  -1   1  -1   1  -1  -1
  1   1  -1   1  -1   1  -1   1
  1   1  -1   1  -1   1   1  -1
  1   1  -1   1  -1   1   1   1
  1   1  -1   1   1  -1  -1  -1
  1   1  -1   1   1  -1  -1   1
  1   1  -1   1   1  -1   1  -1
  1   1  -1   1   1  -1   1   1
  1   1  -1   1   1   1  -1  -1
  1   1  -1   1   1   1  -1   1
  1   1  -1   1   1   1   1  -1
  1   1  -1   1   1   1   1   1
  1   1   1   1  -1  -1  -1  -1
  1   1   1   1  -1  -1  -1   1
  1   1   1   1  -1  -1   1  -1
  1   1   1   1  -1  -1   1   1
  1   1   1   1  -1   1  -1  -1
  1   1   1   1  -1   1  -1   1
  1   1   1   1  -1   1   1  -1
  1   1   1   1  -1   1   1   1
  1   1   1   1   1   1  -1  -1
  1   1   1   1   1   1  -1   1
  1   1   1   1   1   1   1   1
  1   1   1   1   1   1   1  -1
  1   1   1   1   1  -1   1   1
  1   1   1   1   1  -1   1  -1
```

1	1	1	1	1	−1	−1	1
1	1	1	1	1	−1	−1	−1
1	1	1	−1	1	1	1	1
1	1	1	−1	1	1	1	−1
1	1	1	−1	1	1	−1	1
1	1	1	−1	1	1	−1	−1
1	1	1	−1	1	−1	1	1
1	1	1	−1	1	−1	1	−1
1	1	1	−1	1	−1	−1	1
1	1	1	−1	1	−1	−1	−1
1	1	1	−1	−1	1	1	1
1	1	1	−1	−1	1	1	−1
1	1	1	−1	−1	1	−1	1
1	1	1	−1	−1	1	−1	−1
1	1	1	−1	−1	−1	1	1
1	1	1	−1	−1	−1	1	−1
1	1	1	−1	−1	−1	−1	1
1	1	1	−1	−1	−1	−1	−1
1	−1	1	1	1	1	1	1
1	−1	1	1	1	1	1	−1
1	−1	1	1	1	1	−1	1
1	−1	1	1	1	1	−1	−1
1	−1	1	1	1	−1	1	1
1	−1	1	1	1	−1	1	−1
1	−1	1	1	1	−1	−1	1
1	−1	1	1	1	−1	−1	−1
1	−1	1	1	−1	1	1	1
1	−1	1	1	−1	1	1	−1
1	−1	1	1	−1	1	−1	1
1	−1	1	1	−1	1	−1	−1
1	−1	1	1	−1	−1	1	1
1	−1	1	1	−1	−1	1	−1
1	−1	1	1	−1	−1	−1	1
1	−1	1	1	−1	−1	−1	−1
1	−1	1	−1	1	1	1	1
1	−1	1	−1	1	1	1	−1
1	−1	1	−1	1	1	−1	1
1	−1	1	−1	1	1	−1	−1
1	−1	1	−1	1	−1	1	1
1	−1	1	−1	1	−1	1	−1
1	−1	1	−1	1	−1	−1	1
1	−1	1	−1	1	−1	−1	−1
1	−1	1	−1	−1	1	1	1
1	−1	1	−1	−1	1	1	−1
1	−1	1	−1	−1	1	−1	1
1	−1	1	−1	−1	1	−1	−1
1	−1	1	−1	−1	−1	1	1
1	−1	1	−1	−1	−1	1	−1
1	−1	1	−1	−1	−1	−1	1
1	−1	1	−1	−1	−1	−1	−1
1	−1	−1	1	1	1	1	1
1	−1	−1	1	1	1	1	−1
1	−1	−1	1	1	1	−1	1
1	−1	−1	1	1	1	−1	−1
1	−1	−1	1	1	−1	1	1
1	−1	−1	1	1	−1	1	−1
1	−1	−1	1	1	−1	−1	1
1	−1	−1	1	1	−1	−1	−1
1	−1	−1	1	−1	1	1	1
1	−1	−1	1	−1	1	1	−1
1	−1	−1	1	−1	1	−1	1
1	−1	−1	1	−1	1	−1	−1
1	−1	−1	1	−1	−1	1	1
1	−1	−1	1	−1	−1	1	−1

```
  1 -1 -1  1 -1 -1 -1  1
  1 -1 -1  1 -1 -1 -1 -1
  1 -1 -1 -1  1  1  1  1
  1 -1 -1 -1  1  1  1 -1
  1 -1 -1 -1  1  1 -1  1
  1 -1 -1 -1  1  1 -1 -1
  1 -1 -1 -1  1 -1  1  1
  1 -1 -1 -1  1 -1  1 -1
  1 -1 -1 -1  1 -1 -1  1
  1 -1 -1 -1  1 -1 -1 -1
  1 -1 -1 -1 -1  1  1  1
  1 -1 -1 -1 -1  1  1 -1
  1 -1 -1 -1 -1  1 -1  1
  1 -1 -1 -1 -1  1 -1 -1
  1 -1 -1 -1 -1 -1  1  1
  1 -1 -1 -1 -1 -1  1 -1
  1 -1 -1 -1 -1 -1 -1  1
  1 -1 -1 -1 -1 -1 -1 -1
end

~~~~~~ cddlib response

H-representation
begin
 14 8 real
  1  0  0  0  0  0  0  1
  1  0  0  0  0  0  1  0
  1  0  0  0  0  1  0  0
  1  0  0  0  1  0  0  0
  1  0  0  1  0  0  0  0
  1  0  1  0  0  0  0  0
  1  1  0  0  0  0  0  0
  1  0  0  0  0  0  0 -1
  1  0  0  0  0  0 -1  0
  1  0  0  0  0 -1  0  0
  1  0  0  0 -1  0  0  0
  1  0  0 -1  0  0  0  0
  1  0 -1  0  0  0  0  0
  1 -1  0  0  0  0  0  0
end
```

4. Min-max calculation for the quantum bounds of two-two-state particles

```
(* ~~~~~~~~~~~~~~~~~~~~~~~~~~~~~~~~~~~~~~~~~~~~~~~~~~~~~~~~~~~~~~~~~~~~~~~~~~~~~~~ *)
(* ~~~~~~~~~~~~~~~~~~ Start Mathematica Code ~~~~~~~~~~~~~~~~~~~~~~~~~~~~~~~~~~~~ *)
(* ~~~~~~~~~~~~~~~~~~~~~~~~~~~~~~~~~~~~~~~~~~~~~~~~~~~~~~~~~~~~~~~~~~~~~~~~~~~~~~~ *)

(* old stuff

<<Algebra`ReIm`

Normalize[z_]:= z/Sqrt[z.Conjugate[z]];     *)

(*Definition of "my" Tensor Product*)
(*a,b are nxn and mxm-matrices*)

MyTensorProduct[a_, b_] :=
  Table[
   a[[Ceiling[s/Length[b]], Ceiling[t/Length[b]]]]*
```

```
    b[[s - Floor[(s - 1)/Length[b]]*Length[b],
      t - Floor[(t - 1)/Length[b]]*Length[b]]], {s, 1,
    Length[a]*Length[b]}, {t, 1, Length[a]*Length[b]}];


(*Definition of the Tensor Product between two vectors*)

TensorProductVec[x_, y_] :=
  Flatten[Table[
    x[[i]] y[[j]], {i, 1, Length[x]}, {j, 1, Length[y]}]];


(*Definition of the Dyadic Product*)

DyadicProductVec[x_] :=
  Table[x[[i]] Conjugate[x[[j]]], {i, 1, Length[x]}, {j, 1,
    Length[x]}];

(*Definition of the sigma matrices*)


vecsig[r_, tt_, p_] :=
 r*{{Cos[tt], Sin[tt] Exp[-I p]}, {Sin[tt] Exp[I p], -Cos[tt]}}

(*Definition of some vectors*)

BellBasis = (1/Sqrt[2]) {{1, 0, 0, 1}, {0, 1, 1, 0}, {0, 1, -1,
     0}, {1, 0, 0, -1}};

Basis = {{1, 0, 0, 0}, {0, 1, 0, 0}, {0, 0, 1, 0}, {0, 0, 0, 1}};



(*~~~~~~~~~~~~~~~~~~~~~~~~~  2  PARTICLES ~~~~~~~~~~~~~~~~~~~~~~~~~~~~~~~~~~~~~~*)

(*~~~~~~~~~~~~~~~~~~~~~~~~~  2  State System ~~~~~~~~~~~~~~~~~~~~~~~~~~~~~~~~~~~~~

% ~~~~~~~~~~~~~~~   2 x 2
% ~~~~~~~~~~~~~~~   2 x 2
% ~~~~~~~~~~~~~~~   2 x 2
% ~~~~~~~~~~~~~~~   2 x 2
% ~~~~~~~~~~~~~~~   2 x 2
% ~~~~~~~~~~~~~~~   2 x 2

*)


(*Definition of singlet state*)
vp = {1,0};
vm = {0,1};
psi2s = (1/Sqrt[2])*(TensorProductVec[vp, vm] -
    TensorProductVec[vm, vp])

DyadicProductVec[psi2s]

(*Definition of operators*)

(* Definition of one-particle operator *)

M2X = (1/2) {{0, 1}, {1, 0}};
M2Y = (1/2) {{0, -I}, {I, 0}};
M2Z = (1/2) {{1, 0}, {0, -1}};
```

```
Eigenvectors[M2X]
Eigenvectors[M2Y]
Eigenvectors[M2Z]

S2[t_, p_] := FullSimplify[M2X *Sin[t] Cos[p] + M2Y *Sin[t] Sin[p] + M2Z *Cos[t]]

FullSimplify[S2[\[Theta], \[Phi]]] // MatrixForm

FullSimplify[ComplexExpand[S2[Pi/2, 0]]] // MatrixForm
FullSimplify[ComplexExpand[S2[Pi/2, Pi/2]]] // MatrixForm
FullSimplify[ComplexExpand[S2[0, 0]]] // MatrixForm

Assuming[{0 <= \[Theta] <= Pi, 0 <= \[Phi] <= 2 Pi}, FullSimplify[Eigensystem[S2[\[Theta], \[
    Phi]]], {Element[\[Theta], Reals],
  Element[\[Phi], Reals]}]]



FullSimplify[
 Normalize[
  Eigenvectors[S2[\[Theta], \[Phi]]][[1]]], {Element[\[Theta], Reals],
   Element[\[Phi], Reals]}]

ES2M[\[Theta]_,\[Phi]_] := {-E^(-I \[Phi]) Tan[\[Theta]/2], 1}*Cos[\[Theta]/2]*E^(I \[Phi]/2)
ES2P[\[Theta]_,\[Phi]_] := {E^(-I \[Phi]) Cot[\[Theta]/2], 1}*Sin[\[Theta]/2]*E^(I \[Phi]/2)

FullSimplify[ES2M[\[Theta],\[Phi]] .Conjugate[ES2M [\[Theta],\[Phi]]], {Element[\[Theta],
    Reals],
  Element[\[Phi], Reals]}]
FullSimplify[ES2P[\[Theta],\[Phi]] .Conjugate[ES2P [\[Theta],\[Phi]]], {Element[\[Theta],
    Reals],
  Element[\[Phi], Reals]}]
FullSimplify[ES2P[\[Theta],\[Phi]] .Conjugate[ES2M[\[Theta],\[Phi]]], {Element[\[Theta], Reals
    ],
  Element[\[Phi], Reals]}]


ProjectorES2M[\[Theta]_,\[Phi]_] := FullSimplify[DyadicProductVec[ES2M[\[Theta],\[Phi]]], {
    Element[\[Theta], Reals],
  Element[\[Phi], Reals]}]
ProjectorES2P[\[Theta]_,\[Phi]_] := FullSimplify[DyadicProductVec[ES2P[\[Theta],\[Phi]]], {
    Element[\[Theta], Reals],
  Element[\[Phi], Reals]}]

 ProjectorES2M[\[Theta],\[Phi]] //MatrixForm
 ProjectorES2P[\[Theta],\[Phi]] //MatrixForm


(* verification of spectral form *)

FullSimplify[(-1/2)ProjectorES2M[\[Theta],\[Phi]] + (1/2)ProjectorES2P[\[Theta],\[Phi]], {
    Element[\[Theta], Reals],
  Element[\[Phi], Reals]}]


SingleParticleSpinOneHalfeObservable[x_, p_] :=   FullSimplify[(1/2) (IdentityMatrix[2] +
    vecsig[1, x, p])] ;

SingleParticleSpinOneHalfeObservable[\[Theta], \[Phi]] // MatrixForm

Eigensystem[FullSimplify[SingleParticleSpinOneHalfeObservable[x, p]]]


(*Definition of single operators for occurrence of spin up*)
```

```
SingleParticleProjector2first[x_, p_, pm_] :=   MyTensorProduct[1/2 (IdentityMatrix[2] + pm*
    vecsig[1, x, p]),  IdentityMatrix[2]]

SingleParticleProjector2second[x_, p_, pm_] :=  MyTensorProduct[IdentityMatrix[2], 1/2 (
    IdentityMatrix[2] + pm*vecsig[1, x, p])]



(*Definition of two-particle joint operator for occurrence of spin up \
and down*)

JointProjector2[x1_, x2_, p1_, p2_, pm1_, pm2_] :=  MyTensorProduct[1/2 (IdentityMatrix[2] +
    pm1*vecsig[1, x1, p1]),  1/2 (IdentityMatrix[2] + pm2*vecsig[1, x2, p2])]


(*Definition of probabilities*)


(*Probability of concurrence of two equal events for two-particle \
probability in singlet Bell state for occurrence of spin up*)

JointProb2s[x1_, x2_, p1_, p2_, pm1_, pm2_] :=
 FullSimplify[
  Tr[DyadicProductVec[psi2s].JointProjector2[x1, x2, p1, p2, pm1,
     pm2]]]

JointProb2s[x1, x2, p1, p2, pm1, pm2]

JointProb2s[x1, x2, p1, p2, pm1, pm2] // TeXForm

(*sum of joint probabilities add up to one*)

FullSimplify[
 Sum[JointProb2s[x1, x2, p1, p2, pm1, pm2], {pm1, -1, 1, 2}, {pm2, -1,
    1, 2}]]

(*Probability of concurrence of two equal events*)

P2Es[x1_, x2_, p1_, p2_] =
  FullSimplify[
   Sum[UnitStep[pm1*pm2]*
     JointProb2s[x1, x2, p1, p2, pm1, pm2], {pm1, -1, 1, 2}, {pm2, -1,
      1, 2}]];

P2Es[x1, x2, p1, p2]

(*Probability of concurrence of two non-equal events*)

P2NEs[x1_, x2_, p1_, p2_] =
  FullSimplify[
   Sum[UnitStep[-pm1*pm2]*
     JointProb2s[x1, x2, p1, p2, pm1, pm2], {pm1, -1, 1, 2}, {pm2, -1,
      1, 2}]];

P2NEs[x1, x2, p1, p2]

(*Expectation function*)

Expectation2s[x1_, x2_, p1_, p2_] =
 FullSimplify[P2Es[x1, x2, p1, p2] - P2NEs[x1, x2, p1, p2]]
```

```
(* ~~~~~~~~~~~~~~~~~~~~~~ Min-Max calculation of the quantum correlation function
    ~~~~~~~~~~~~~~~~~~~~~~ *)

JointExpectation2[t1_, t2_, p1_, p2_] := MyTensorProduct[ 2 * S2[t1, p1] , 2 * S2[t2, p2] ]


FullSimplify[
 Eigensystem[
 JointExpectation2[t1 , t2 , p1 , p2 ]  ]]    // MatrixForm

FullSimplify[
 Eigensystem[
 DyadicProductVec[psi2s]. JointExpectation2[t1, t2, p1 , p2 ] . DyadicProductVec[psi2s]  ]]
    // MatrixForm

FullSimplify[
 Eigensystem[
 JointExpectation2[Pi/2 , Pi/2 , p1 , p2 ]  ]]         // MatrixForm

FullSimplify[
  Eigensystem[
   DyadicProductVec[psi2s].JointExpectation2[Pi/2, Pi/2, p1,  p2 ].DyadicProductVec[psi2s]]]
      // MatrixForm


psi2mp = (1/Sqrt[2])*(TensorProductVec[vp, vm] +
    TensorProductVec[vm, vp])

psi2mm = (1/Sqrt[2])*(TensorProductVec[vp, vp] -
    TensorProductVec[vm, vm])

psi2pp = (1/Sqrt[2])*(TensorProductVec[vp, vp] +
    TensorProductVec[vm, vm])


FullSimplify[
  Eigensystem[
   DyadicProductVec[psi2mp].JointExpectation2[Pi/2, Pi/2, p1,
     p2].DyadicProductVec[psi2mp]]] // MatrixForm

FullSimplify[
  Eigensystem[
   DyadicProductVec[psi2mm].JointExpectation2[Pi/2, Pi/2, p1,
     p2].DyadicProductVec[psi2mm]]] // MatrixForm

FullSimplify[
  Eigensystem[
   DyadicProductVec[psi2pp].JointExpectation2[Pi/2, Pi/2, p1,
     p2].DyadicProductVec[psi2pp]]] // MatrixForm

(* ~~~~~~~~~~~~~~~~~~~~~~ Min-Max calculation of the Tsirelson bound ~~~~~~~~~~~~~~~~~~~~~~
    *)

JointProjector2Red[ p1_, p2_, pm1_, pm2_] :=  JointProjector2[ Pi/2 , Pi/2 , p1, p2, pm1, pm2]

FullSimplify[ JointProjector2Red[ p1 , p2 , pm1 , pm2 ]]

(* ~~~~~~~~~~~~~~~~~~~~~~~~ plausibility check *)

JointProb2sRed[p1_, p2_, pm1_, pm2_] :=
 FullSimplify[
  Tr[DyadicProductVec[psi2s].JointProjector2Red[p1, p2, pm1, pm2]]]

JointProb2sRed[p1, p2, pm1, pm2]
```

```
FullSimplify[
 JointProb2sRed[p1, p2, 1, 1] + JointProb2sRed[p1, p2, -1, -1] -
  JointProb2sRed[p1, p2, -1, 1] - JointProb2sRed[p1, p2, 1, -1]]

(* ~~~~~~~~~~~~~~~~~~~~~~~~~ end plausibility check *)


TwoParticleExpectationsRed[ p1_, p2_] := JointProjector2Red[ p1, p2, 1, 1]  +
    JointProjector2Red[ p1, p2, -1, -1] -
                                         JointProjector2Red[ p1, p2, -1, 1] -
                                             JointProjector2Red[ p1, p2, 1, -1]


(* ~~~~~~~~~~~~~~~~~~~~~~~~~ plausibility check *)

FullSimplify[ Tr[DyadicProductVec[psi2s].TwoParticleExpectationsRed[A1, B1]] ]

(* ~~~~~~~~~~~~~~~~~~~~~~~~~ end plausibility check *)

TwoParticleExpectationsRed[A1, B1] // MatrixForm
TwoParticleExpectationsRed[A1, B1] // TeXForm

Eigenvalues[
 ComplexExpand[
  TwoParticleExpectationsRed[A1, B1] +
   TwoParticleExpectationsRed[A2, B1] +
   TwoParticleExpectationsRed[A1, B2] -
   TwoParticleExpectationsRed[A2, B2] ]]

FullSimplify[
 Eigenvalues[
  ComplexExpand[
   TwoParticleExpectationsRed[A1, B1] +
    TwoParticleExpectationsRed[A2, B1] +
    TwoParticleExpectationsRed[A1, B2] -
    TwoParticleExpectationsRed[A2, B2] ]]]


FullSimplify[
   TwoParticleExpectationsRed[A1, B1] +
    TwoParticleExpectationsRed[A2, B1] +
    TwoParticleExpectationsRed[A1, B2] -
    TwoParticleExpectationsRed[A2, B2] ]

(*  observables along psi_singlet *)


Eigenvalues[
 ComplexExpand[
  DyadicProductVec[
    psi2s].( TwoParticleExpectationsRed[A1, B1] +
     TwoParticleExpectationsRed[A2, B1] +
     TwoParticleExpectationsRed[A1, B2] -
     TwoParticleExpectationsRed[A2, B2]).DyadicProductVec[psi2s]]]

FullSimplify[
 TrigExpand[
  Eigenvalues[
   ComplexExpand[
    DyadicProductVec[
      psi2s].( TwoParticleExpectationsRed[0, Pi/4] +
       TwoParticleExpectationsRed[Pi/2, Pi/4] +
       TwoParticleExpectationsRed[0, -Pi/4] -
```

```
        TwoParticleExpectationsRed[Pi/2, -Pi/4]).DyadicProductVec[
       psi2s]]]]]

(*  observables along psi_+ *)


Eigenvalues[
 ComplexExpand[
  DyadicProductVec[
    psi2mp].(TwoParticleExpectationsRed[A1, B1] +
     TwoParticleExpectationsRed[A2, B1] +
     TwoParticleExpectationsRed[A1, B2] -
     TwoParticleExpectationsRed[A2, B2]).DyadicProductVec[psi2mp]]]

FullSimplify[
 TrigExpand[
  Eigenvalues[
   ComplexExpand[
    DyadicProductVec[
      psi2mp].(TwoParticleExpectationsRed[0, Pi/4] +
       TwoParticleExpectationsRed[Pi/2, Pi/4] +
       TwoParticleExpectationsRed[0, -Pi/4] -
       TwoParticleExpectationsRed[Pi/2, -Pi/4]).DyadicProductVec[
      psi2mp]]]]]

(*** observables along phi_+ ***)


Eigenvalues[
 ComplexExpand[
  DyadicProductVec[
    psi2mm].(TwoParticleExpectationsRed[A1, B1] +
     TwoParticleExpectationsRed[A2, B1] +
     TwoParticleExpectationsRed[A1, B2] -
     TwoParticleExpectationsRed[A2, B2]).DyadicProductVec[psi2mm]]]

FullSimplify[
 TrigExpand[
  Eigenvalues[
   ComplexExpand[
    DyadicProductVec[
      psi2mm].(TwoParticleExpectationsRed[0, -Pi/4] +
       TwoParticleExpectationsRed[Pi/2, -Pi/4] +
       TwoParticleExpectationsRed[0, Pi/4] -
       TwoParticleExpectationsRed[Pi/2, Pi/4]).DyadicProductVec[
      psi2mm]]]]]


(*** observables along phi_+ ***)


Eigenvalues[
 ComplexExpand[
  DyadicProductVec[
    psi2pp].(TwoParticleExpectationsRed[A1, B1] +
     TwoParticleExpectationsRed[A2, B1] +
     TwoParticleExpectationsRed[A1, B2] -
     TwoParticleExpectationsRed[A2, B2]).DyadicProductVec[psi2pp]]]

FullSimplify[
 TrigExpand[
  Eigenvalues[
   ComplexExpand[
    DyadicProductVec[
```

```
      psi2pp].(TwoParticleExpectationsRed[0, -Pi/4] +
       TwoParticleExpectationsRed[Pi/2, -Pi/4] +
       TwoParticleExpectationsRed[0, Pi/4] -
       TwoParticleExpectationsRed[Pi/2, Pi/4]).DyadicProductVec[
      psi2pp]]]]]
```

5. Min-max calculation for the quantum bounds of two three-state particles

```
(* ~~~~~~~~~~~~~~~~~~~~~~~~~~~~~~~~~~~~~~~~~~~~~~~~~~~~~~~~~~~~~~~~~~~~~~~~~~~~ *)
(* ~~~~~~~~~~~~~~~~~~Start Mathematica Code~~~~~~~~~~~~~~~~~~~~~~~~~~~~~~~~~~~~~ *)
(* ~~~~~~~~~~~~~~~~~~~~~~~~~~~~~~~~~~~~~~~~~~~~~~~~~~~~~~~~~~~~~~~~~~~~~~~~~~~~ *)

(* old stuff

<<Algebra`ReIm`

Normalize[z_]:= z/Sqrt[z.Conjugate[z]];     *)

(*Definition of "my" Tensor Product*)
(*a,b are nxn and mxm-matrices*)

MyTensorProduct[a_, b_] :=
  Table[
   a[[Ceiling[s/Length[b]], Ceiling[t/Length[b]]]]*
    b[[s - Floor[(s - 1)/Length[b]]*Length[b],
      t - Floor[(t - 1)/Length[b]]*Length[b]]], {s, 1,
    Length[a]*Length[b]}, {t, 1, Length[a]*Length[b]}];


(*Definition of the Tensor Product between two vectors*)

TensorProductVec[x_, y_] :=
  Flatten[Table[
    x[[i]] y[[j]], {i, 1, Length[x]}, {j, 1, Length[y]}]];


(*Definition of the Dyadic Product*)

DyadicProductVec[x_] :=
  Table[x[[i]] Conjugate[x[[j]]], {i, 1, Length[x]}, {j, 1,
    Length[x]}];

(*Definition of the sigma matrices*)


vecsig[r_, tt_, p_] :=
 r*{{Cos[tt], Sin[tt] Exp[-I p]}, {Sin[tt] Exp[I p], -Cos[tt]}}

(*Definition of some vectors*)

BellBasis = (1/Sqrt[2]) {{1, 0, 0, 1}, {0, 1, 1, 0}, {0, 1, -1,
     0}, {1, 0, 0, -1}};

Basis = {{1, 0, 0, 0}, {0, 1, 0, 0}, {0, 0, 1, 0}, {0, 0, 0, 1}};


 (*~~~~~~~~~~~~~~~~~~~~~~~~~ 3 State System ~~~~~~~~~~~~~~~~~~~~~~~~~~~~~~~~~~~~~~

% ~~~~~~~~~~~~~~~  2 x 3
% ~~~~~~~~~~~~~~~  2 x 3
```

```
% ~~~~~~~~~~~~~~~   2 x 3
% ~~~~~~~~~~~~~~~   2 x 3
% ~~~~~~~~~~~~~~~   2 x 3
% ~~~~~~~~~~~~~~~   2 x 3
% ~~~~~~~~~~~~~~~   2 x 3
% ~~~~~~~~~~~~~~~   2 x 3

*)



(*Definition of operators*)

(* Definition of one-particle operator *)

M3X = (1/Sqrt[2]) {{0, 1, 0}, {1, 0, 1},{0, 1, 0}};
M3Y = (1/Sqrt[2]) {{0, -I, 0}, {I, 0, -I}, {0, I, 0}};
M3Z =  {{1, 0, 0}, {0, 0, 0},{0, 0, -1}};


Eigenvectors[M3X]
Eigenvectors[M3Y]
Eigenvectors[M3Z]

S3[t_, p_] := M3X *Sin[t] Cos[p] + M3Y *Sin[t] Sin[p] + M3Z *Cos[t]

FullSimplify[S3[\[Theta], \[Phi]]] // MatrixForm

FullSimplify[ComplexExpand[S3[Pi/2, 0]]] // MatrixForm
FullSimplify[ComplexExpand[S3[Pi/2, Pi/2]]] // MatrixForm
FullSimplify[ComplexExpand[S3[0, 0]]] // MatrixForm

Assuming[{0 <= \[Theta] <= Pi, 0 <= \[Phi] <= 2 Pi}, FullSimplify[Eigensystem[S3[\[Theta], \[
    Phi]]], {Element[\[Theta], Reals],
  Element[\[Phi], Reals]}]]



FullSimplify[ComplexExpand[
 Normalize[
  Eigenvectors[S3[\[Theta], \[Phi]]][[1]]], {Element[\[Theta], Reals],
   Element[\[Phi], Reals]}]]

ES3M[\[Theta]_,\[Phi]_] := FullSimplify[ ComplexExpand[
 Normalize[
  Eigenvectors[S3[\[Theta], \[Phi]]][[1]]]*E^(I \[Phi])  , {Element[\[Theta], Reals], Element
      [\[Phi], Reals]}]]

ES3M[\[Theta],\[Phi]]


ES3P[\[Theta]_,\[Phi]_] := FullSimplify[ComplexExpand[
 Normalize[
  Eigenvectors[S3[\[Theta], \[Phi]]][[2]]]*E^(I \[Phi])  , {Element[\[Theta], Reals], Element
      [\[Phi], Reals]}]]

ES3P[\[Theta],\[Phi]]

ES30[\[Theta]_,\[Phi]_] := FullSimplify[ComplexExpand[
 Normalize[
  Eigenvectors[S3[\[Theta], \[Phi]]][[3]]]*E^(I \[Phi])  , {Element[\[Theta], Reals], Element
      [\[Phi], Reals]}]]

ES30[\[Theta],\[Phi]]
```

```
FullSimplify[ES3M[\[Theta],\[Phi]] .Conjugate[ES3M [\[Theta],\[Phi]]], {Element[\[Theta],
    Reals],
  Element[\[Phi], Reals]}]
FullSimplify[ES3P[\[Theta],\[Phi]] .Conjugate[ES3P [\[Theta],\[Phi]]], {Element[\[Theta],
    Reals],
  Element[\[Phi], Reals]}]
FullSimplify[ES30[\[Theta],\[Phi]] .Conjugate[ES30 [\[Theta],\[Phi]]], {Element[\[Theta],
    Reals],
  Element[\[Phi], Reals]}]
FullSimplify[ES3P[\[Theta],\[Phi]] .Conjugate[ES3M[\[Theta],\[Phi]]], {Element[\[Theta], Reals
    ],
  Element[\[Phi], Reals]}]
FullSimplify[ES3P[\[Theta],\[Phi]] .Conjugate[ES30[\[Theta],\[Phi]]], {Element[\[Theta], Reals
    ],
  Element[\[Phi], Reals]}]
FullSimplify[ES30[\[Theta],\[Phi]] .Conjugate[ES3M[\[Theta],\[Phi]]], {Element[\[Theta], Reals
    ],
  Element[\[Phi], Reals]}]


ProjectorES30[\[Theta]_,\[Phi]_] := FullSimplify[ComplexExpand[DyadicProductVec[ES30[\[Theta
    ],\[Phi]]]], {Element[\[Theta], Reals],
  Element[\[Phi], Reals]}]]
ProjectorES3M[\[Theta]_,\[Phi]_] := FullSimplify[ComplexExpand[DyadicProductVec[ES3M[\[Theta
    ],\[Phi]]]], {Element[\[Theta], Reals],
  Element[\[Phi], Reals]}]]
ProjectorES3P[\[Theta]_,\[Phi]_] := FullSimplify[ComplexExpand[DyadicProductVec[ES3P[\[Theta
    ],\[Phi]]]], {Element[\[Theta], Reals],
  Element[\[Phi], Reals]}]]

 ProjectorES30[\[Theta],\[Phi]] //MatrixForm
 ProjectorES3M[\[Theta],\[Phi]] //MatrixForm
 ProjectorES3P[\[Theta],\[Phi]] //MatrixForm

ProjectorES30[\[Theta], \[Phi]] // MatrixForm // TeXForm
ProjectorES3M[\[Theta], \[Phi]] // MatrixForm // TeXForm
ProjectorES3P[\[Theta], \[Phi]] // MatrixForm // TeXForm

(* verification of spectral form *)

FullSimplify[0 * ProjectorES30[\[Theta],\[Phi]] + (-1) * ProjectorES3M[\[Theta],\[Phi]] +
    (+1) * ProjectorES3P[\[Theta],\[Phi]], {Element[\[Theta], Reals],
  Element[\[Phi], Reals]}] //MatrixForm


(*  ~~~~~~~~~~~~~~~~~~ general operator ~~~~~~~~~~~~~~~~~~~~~~  *)

Operator3GEN[\[Theta]_,\[Phi]_] := FullSimplify[LM * ProjectorES3M[\[Theta],\[Phi]] + L0 *
    ProjectorES30[\[Theta],\[Phi]] + LP * ProjectorES3P[\[Theta],\[Phi]], {Element[\[Theta],
    Reals], Element[\[Phi], Reals]}];

Operator3GEN[\[Theta],\[Phi]]

JointProjector3GEN[x1_, x2_, p1_, p2_] :=  MyTensorProduct[Operator3GEN[x1,p1],Operator3GEN[x2
    ,p2]];

v3p = {1,0,0};
v30 = {0,1,0};
v3m = {0,0,1};

psi3s = (1/Sqrt[3])*(-TensorProductVec[v30, v30] + TensorProductVec[v3m, v3p] +
    TensorProductVec[v3p, v3m])
```

```
Expectation3sGEN[x1_, x2_, p1_, p2_] := FullSimplify[ Tr[DyadicProductVec[psi3s].
    JointProjector3GEN[x1, x2, p1, p2]]];

Expectation3sGEN[x1, x2, p1, p2]


Ex3[LM_,L0_,LP_,x1_,x2_,p1_,p2_]:=FullSimplify[1/192 (24 L0^2 + 40 L0 (LM + LP) + 22 (LM + LP)
    ^2 -
   32 (LM - LP)^2 Cos[x1] Cos[x2] +
   2 (-2 L0 + LM + LP)^2 Cos[
     2 x2] ((3 + Cos[2 (p1 - p2)]) Cos[2 x1] + 2 Sin[p1 - p2]^2) +
   2 (-2 L0 + LM + LP)^2 (Cos[2 (p1 - p2)] +
      2 Cos[2 x1] Sin[p1 - p2]^2) -
   32 (LM - LP)^2 Cos[p1 - p2] Sin[x1] Sin[x2] +
   8 (-2 L0 + LM + LP)^2 Cos[p1 - p2] Sin[2 x1] Sin[2 x2])];

Ex3[-1,0,1,x1,x2,p1,p2]



(* ~~~~~~~~~~~ natural spin observables ~~~~~~~~~~~~~~~~~~~~~ *)



JointProjector3NAT[x1_, x2_, p1_, p2_] :=  MyTensorProduct[S3[x1,p1],S3[x2,p2]];

Expectation3sNAT[x1_, x2_, p1_, p2_] := FullSimplify[ Tr[DyadicProductVec[psi3s].
    JointProjector3NAT[x1, x2, p1, p2]]];

Expectation3sNAT[x1, x2, p1, p2]



(* ~~~~~~~~~~~ Kochen-Specker observables ~~~~~~~~~~~~~~~~~~~~~ *)


(*
S3[t_, p_] := M3X *Sin[t] Cos[p] + M3Y *Sin[t] Sin[p] + M3Z *Cos[t]

MM3X[ \[Alpha]_ ] := FullSimplify[S3[Pi/2, \[Alpha]]];
MM3Y[ \[Alpha]_ ] := FullSimplify[S3[Pi/2, \[Alpha]+Pi/2]];
MM3Z[ \[Alpha]_ ] := FullSimplify[S3[0, 0]];

SKS[ \[Alpha]_ ] := FullSimplify[ MM3X[\[Alpha]].MM3X[\[Alpha]] + MM3Y[\[Alpha]].MM3Y[\[Alpha
    ]] + MM3Z[\[Alpha]].MM3Z[\[Alpha]] ];

FullSimplify[SKS[ \[Alpha] ]] // MatrixForm

FullSimplify[ComplexExpand[SKS[ 0]]] // MatrixForm
FullSimplify[ComplexExpand[SKS[ Pi/2]]] // MatrixForm

Assuming[{0 <= \[Theta] <= Pi, 0 <= \[Phi] <= 2 Pi}, FullSimplify[Eigensystem[SKS[ \[Alpha]
    ]], {Element[\[Alpha], Reals]}]]

*)

Ex3[1, 0, 1, \[Theta]1, \[Theta]2, \[CurlyPhi]1, \[CurlyPhi]2]

Ex3[0, 1, 0, \[Theta]1, \[Theta]2, \[CurlyPhi]1, \[CurlyPhi]2]

Ex3[1, 0, 1, Pi/2, Pi/2, \[CurlyPhi]1, \[CurlyPhi]2]

Ex3[0, 1, 0, Pi/2, Pi/2, \[CurlyPhi]1, \[CurlyPhi]2]
```

```
Ex3[1, 0, 1, \[Theta]1, \[Theta]2, 0, 0]

Ex3[0, 1, 0, \[Theta]1, \[Theta]2, 0, 0]



(* min-max computation *)

(* define dichotomic operator based on spin-1  expectation value , take \[Phi] = Pi/2 *)

(* old, invalid parameterization
A[ \[Theta]1_ , \[Theta]2_  ] :=   MyTensorProduct[ S3[\[Theta]1, Pi/2] ,  S3[\[Theta]2, Pi/2]
     ]

(* Form the Klyachko-Can-Biniciogolu-Shumovsky operator  *)

T[\[Theta]1_, \[Theta]3_, \[Theta]5_, \[Theta]7_, \[Theta]9_] :=
 A[\[Theta]1,\[Theta]3] + A[\[Theta]3,\[Theta]5] +
  A[\[Theta]5,\[Theta]7] + A[\[Theta]7,\[Theta]9] +
  A[\[Theta]9,\[Theta]1]


FullSimplify[
 Eigenvalues[
  FullSimplify[
  T[\[Theta]1, \[Theta]3, \[Theta]5, \[Theta]7, \[Theta]9]]]]

FullSimplify[
 Eigenvalues[
  T[2 Pi/5 , 4 Pi/5, 6 Pi/5, 8 Pi/5, 2 Pi]]]

 *)

A[ \[Theta]1_ , \[Theta]2_ ,\[CurlyPhi]1_, \[CurlyPhi]2_  ] :=   MyTensorProduct[ S3[\[Theta
    ]1, \[CurlyPhi]1] ,  S3[\[Theta]2, \[CurlyPhi]2] ]

(* Form the Klyachko-Can-Biniciogolu-Shumovsky operator  *)

T[\[Theta]1_, \[Theta]3_, \[Theta]5_, \[Theta]7_, \[Theta]9_,\[CurlyPhi]1_, \[CurlyPhi]3_,\[
    CurlyPhi]5_, \[CurlyPhi]7_,\[CurlyPhi]9_] :=
 A[\[Theta]1,\[Theta]3, \[CurlyPhi]1 ,\[CurlyPhi]3] + A[\[Theta]3,\[Theta]5,\[CurlyPhi]3,\[
     CurlyPhi]5] +
  A[\[Theta]5,\[Theta]7,\[CurlyPhi]5,\[CurlyPhi]7] + A[\[Theta]7,\[Theta]9,\[CurlyPhi]7,\[
      CurlyPhi]9] +
  A[\[Theta]9,\[Theta]1,\[CurlyPhi]9,\[CurlyPhi]1]


A1  =  CoordinateTransformData[ "Cartesian" -> "Spherical", "Mapping", {1,0,0  }] ;
A2  =  CoordinateTransformData[ "Cartesian" -> "Spherical", "Mapping", {0,1,0  }] ;
A3  =  (* CoordinateTransformData[ "Cartesian" -> "Spherical", "Mapping", {0,0,1  }] *)
    {1,0,Pi/2} ;
A4  =  CoordinateTransformData[ "Cartesian" -> "Spherical", "Mapping", {1,-1,0 }] ;
A5  =  CoordinateTransformData[ "Cartesian" -> "Spherical", "Mapping", {1,1,0  }] ;
A6  =  CoordinateTransformData[ "Cartesian" -> "Spherical", "Mapping", {1,-1,2 }] ;
A7  =  CoordinateTransformData[ "Cartesian" -> "Spherical", "Mapping", {-1,1,1 }] ;
A8  =  CoordinateTransformData[ "Cartesian" -> "Spherical", "Mapping", {2,1,1  }] ;
A9  =  CoordinateTransformData[ "Cartesian" -> "Spherical", "Mapping", {0,1,-1 }] ;
A10 =  CoordinateTransformData[ "Cartesian" -> "Spherical", "Mapping", {0,1,1  }] ;



 FullSimplify[
```

```
 Eigenvalues[
  FullSimplify[
  T[ A1[[2]],  A3[[2]],  A5[[2]],  A7[[2]],  A9[[2]] , A1[[3]],  A3[[3]],  A5[[3]],  A7[[3]],
      A9[[3]]]]]]



{A1,
A2 ,
A3 ,
A4 ,
A5 ,
A6 ,
A7 ,
A8 ,
A9 ,
A10} //TexForm
```

6. Min-max calculation for two four-state particles

```
(* ~~~~~~~~~~~~~~~~~~~~~~~~~~~~~~~~~~~~~~~~~~~~~~~~~~~~~~~~~~~~~~~~~~~~~~ *)
(* ~~~~~~~~~~~~~~~~~~Start Mathematica Code~~~~~~~~~~~~~~~~~~~~~~~~~~~~~~~ *)
(* ~~~~~~~~~~~~~~~~~~~~~~~~~~~~~~~~~~~~~~~~~~~~~~~~~~~~~~~~~~~~~~~~~~~~~~ *)

(* old stuff

<<Algebra`ReIm`

Normalize[z_]:= z/Sqrt[z.Conjugate[z]];     *)

(*Definition of "my" Tensor Product*)
(*a,b are nxn and mxm-matrices*)

MyTensorProduct[a_, b_] :=
  Table[
   a[[Ceiling[s/Length[b]], Ceiling[t/Length[b]]]]*
    b[[s - Floor[(s - 1)/Length[b]]*Length[b],
      t - Floor[(t - 1)/Length[b]]*Length[b]]], {s, 1,
    Length[a]*Length[b]}, {t, 1, Length[a]*Length[b]}];


(*Definition of the Tensor Product between two vectors*)

TensorProductVec[x_, y_] :=
  Flatten[Table[
    x[[i]] y[[j]], {i, 1, Length[x]}, {j, 1, Length[y]}]];


(*Definition of the Dyadic Product*)

DyadicProductVec[x_] :=
  Table[x[[i]] Conjugate[x[[j]]], {i, 1, Length[x]}, {j, 1,
    Length[x]}];

(*Definition of the sigma matrices*)


vecsig[r_, tt_, p_] :=
 r*{{Cos[tt], Sin[tt] Exp[-I p]}, {Sin[tt] Exp[I p], -Cos[tt]}}
```

```
(*Definition of some vectors*)

BellBasis = (1/Sqrt[2]) {{1, 0, 0, 1}, {0, 1, 1, 0}, {0, 1, -1,
     0}, {1, 0, 0, -1}};

Basis = {{1, 0, 0, 0}, {0, 1, 0, 0}, {0, 0, 1, 0}, {0, 0, 0, 1}};


(*~~~~~~~~~~~~~~~~~~~~~~~~~~ 4 State System ~~~~~~~~~~~~~~~~~~~~~~~~~~~~~~~~~~~~~~~~~~

% ~~~~~~~~~~~~~~~   2 x 4
% ~~~~~~~~~~~~~~~   2 x 4
% ~~~~~~~~~~~~~~~   2 x 4
% ~~~~~~~~~~~~~~~   2 x 4
% ~~~~~~~~~~~~~~~   2 x 4
% ~~~~~~~~~~~~~~~   2 x 4
% ~~~~~~~~~~~~~~~   2 x 4
% ~~~~~~~~~~~~~~~   2 x 4

*)




(*Definition of operators*)

(* Definition of one-particle operator *)

M4X = (1/2) {{0,Sqrt[3],0,0 },{Sqrt[3],0,2,0 },{0,2,0,Sqrt[3] },{0,0,Sqrt[3],0 }};
M4Y = (1/2) {{0,-Sqrt[3]I,0,0 },{Sqrt[3]I,0,-2I,0},{0,2I,0,-Sqrt[3]I},{0,0,Sqrt[3]I,0  } };
M4Z = (1/2) {{3,0,0,0 },{0,1,0,0 },{0,0,-1,0},{0,0,0,-3}};

Eigenvectors[M4X]
Eigenvectors[M4Y]
Eigenvectors[M4Z]

S4[t_, p_] :=  FullSimplify[M4X *Sin[t] Cos[p] + M4Y *Sin[t] Sin[p] + M4Z *Cos[t]];



(*  ~~~~~~~~~~~~~~~~~~~ general operator ~~~~~~~~~~~~~~~~~~~~~~   *)

LM32 =-3/2;
LM12 =-1/2;
LP32 =3/2;
LP12 =1/2;

ES4M32[\[Theta]_, \[Phi]_] :=   FullSimplify[   Assuming[{0 < \[Theta] < Pi, 0 <= \[Phi] <= 2
    Pi},    Normalize[     Eigenvectors[S4[\[Theta], \[Phi]]][[1]]]], {Element[\[Theta],
    Reals], Element[\[Phi], Reals]}];
ES4P32[\[Theta]_, \[Phi]_] :=  FullSimplify[   Assuming[{0 < \[Theta] < Pi, 0 <= \[Phi] <= 2
    Pi},    Normalize[     Eigenvectors[S4[\[Theta], \[Phi]]][[2]]]], {Element[\[Theta],
    Reals], Element[\[Phi], Reals]}];
ES4M12[\[Theta]_, \[Phi]_] :=  FullSimplify[   Assuming[{0 < \[Theta] < Pi, 0 <= \[Phi] <= 2
    Pi},    Normalize[     Eigenvectors[S4[\[Theta], \[Phi]]][[3]]]], {Element[\[Theta],
    Reals], Element[\[Phi], Reals]}];
ES4P12[\[Theta]_, \[Phi]_] :=  FullSimplify[   Assuming[{0 < \[Theta] < Pi, 0 <= \[Phi] <= 2
    Pi},    Normalize[     Eigenvectors[S4[\[Theta], \[Phi]]][[4]]]], {Element[\[Theta],
    Reals], Element[\[Phi], Reals]}];



JointProjector4GEN[x1_, x2_, p1_, p2_] :=  TensorProduct[S4[x1,p1],S4[x2,p2]];
```

```
v4P32 = ES4P32[0,0]
v4P12 = ES4P12[0,0]
v4M12 = ES4M12[0,0]
v4M32 = ES4M32[0,0]



psi4s = (1/2)*(TensorProductVec[v4P32, v4M32]-TensorProductVec[v4M32, v4P32] -
    TensorProductVec[v4P12 , v4M12 ] + TensorProductVec[v4M12 , v4P12 ])


Expectation4sGEN[x1_, x2_, p1_, p2_] := Tr[DyadicProductVec[psi4s].JointProjector4GEN[x1, x2,
    p1, p2]];

FullSimplify[Expectation4sGEN[x1, x2, p1, p2]]


(* ~~~~~~~~ general case ~~~~~~~~~ *)

EPPMM1[L4M32_ , L4M12_ , L4P12_ , L4P32_ ,  \[Theta]_, \[Phi]_] :=   Assuming[{0 < \[Theta] <
    Pi, 0 <= \[Phi] <= 2 Pi}, FullSimplify[
L4M32 * Assuming[{0 < \[Theta] < Pi, 0 <= \[Phi] <= 2 Pi},
  FullSimplify[
   DyadicProductVec[
    ES4M32[\[Theta], \[Phi]]], {Element[\[Theta], Reals],
    Element[\[Phi], Reals]}] ]   + L4M12 *    Assuming[{0 < \[Theta] < Pi, 0 <= \[Phi] <= 2 Pi
        },
  FullSimplify[
   DyadicProductVec[
    ES4M12[\[Theta], \[Phi]]], {Element[\[Theta], Reals],
    Element[\[Phi], Reals]}] ]+
L4P32 * Assuming[{0 < \[Theta] < Pi, 0 <= \[Phi] <= 2 Pi},
  FullSimplify[
   DyadicProductVec[
    ES4P32[\[Theta], \[Phi]]], {Element[\[Theta], Reals],
    Element[\[Phi], Reals]}] ]+
L4P12 * Assuming[{0 < \[Theta] < Pi, 0 <= \[Phi] <= 2 Pi},
  FullSimplify[
   DyadicProductVec[
    ES4P12[\[Theta], \[Phi]]], {Element[\[Theta], Reals],
    Element[\[Phi], Reals]}] ]
]]


EPPMM1[-1,-1,1,1,\[Theta], \[Phi]] //MatrixForm

JointProjector4PPMM1[L4M32_ , L4M12_ , L4P12_ , L4P32_ , x1_, x2_, p1_, p2_] :=  Assuming[{0 <
     \[Theta] < Pi, 0 <= \[Phi] <= 2 Pi},
  FullSimplify[TensorProduct[EPPMM1[L4M32 , L4M12 , L4P12 , L4P32 , x1,p1],EPPMM1[L4M32 , L4M12
      , L4P12 , L4P32 ,x2,p2]], {Element[\[Theta], Reals],
    Element[\[Phi], Reals]}] ];

Expectation4PPMM1[L4M32_ , L4M12_ , L4P12_ , L4P32_ , x1_, x2_, p1_, p2_] := Tr[
    DyadicProductVec[psi4s].JointProjector4PPMM1[L4M32 , L4M12 , L4P12 , L4P32 ,x1, x2, p1, p2
    ]];

FullSimplify[Expectation4PPMM1[-1,-1,1,1,x1, x2, p1, p2]]

Emmpp[x1_ ]= FullSimplify[Expectation4PPMM1[-1, -1, 1, 1, x1, 0, 0, 0]];
Emppm[x1_ ]= FullSimplify[Expectation4PPMM1[-1, 1, 1, -1, x1, 0, 0, 0]];
Empmp[x1_ ]= FullSimplify[Expectation4PPMM1[-1, 1, -1, 1, x1, 0, 0, 0]];
```

```
(*********** minmax calculation *************)

v12  = Normalize [ { 1,0,0,0      } ]  ;
v18  = Normalize [ { 0,1,0,0      } ]  ;
v17  = Normalize [ { 0,0,1,1      } ] ;
v16  = Normalize [ { 0,0,1,-1   } ]  ;
v67  = Normalize [ { 1,-1,0,0   } ]  ;
v69  = Normalize [ { 1,1,-1,-1  } ]  ;
v56  = Normalize [ { 1,1,1,1    } ]  ;
v59  = Normalize [ { 1,-1,1,-1  } ]  ;
v58  = Normalize [ { 1,0,-1,0   } ]  ;
v45  = Normalize [ { 0,1,0,-1   } ]  ;
v48  = Normalize [ { 1,0,1,0    } ]  ;
v47  = Normalize [ { 1,1,-1,1   } ]  ;
v34  = Normalize [ { -1,1,1,1   } ]  ;
v37  = Normalize [ { 1,1,1,-1   } ]  ;
v39  = Normalize [ { 1,0,0,1    } ]  ;
v23  = Normalize [ { 0,1,-1,0   } ]  ;
v29  = Normalize [ { 0,1,1,0    } ]  ;
v28  = Normalize [ { 0,0,0,1      } ]  ;

A12  = 2 * DyadicProductVec[ v12 ] -  IdentityMatrix[4];
A18  = 2 * DyadicProductVec[ v18 ] -  IdentityMatrix[4];
A17  = 2 * DyadicProductVec[ v17 ] -  IdentityMatrix[4];
A16  = 2 * DyadicProductVec[ v16 ] -  IdentityMatrix[4];
A67  = 2 * DyadicProductVec[ v67 ] -  IdentityMatrix[4];
A69  = 2 * DyadicProductVec[ v69 ] -  IdentityMatrix[4];
A56  = 2 * DyadicProductVec[ v56 ] -  IdentityMatrix[4];
A59  = 2 * DyadicProductVec[ v59 ] -  IdentityMatrix[4];
A58  = 2 * DyadicProductVec[ v58 ] -  IdentityMatrix[4];
A45  = 2 * DyadicProductVec[ v45 ] -  IdentityMatrix[4];
A48  = 2 * DyadicProductVec[ v48 ] -  IdentityMatrix[4];
A47  = 2 * DyadicProductVec[ v47 ] -  IdentityMatrix[4];
A34  = 2 * DyadicProductVec[ v34 ] -  IdentityMatrix[4];
A37  = 2 * DyadicProductVec[ v37 ] -  IdentityMatrix[4];
A39  = 2 * DyadicProductVec[ v39 ] -  IdentityMatrix[4];
A23  = 2 * DyadicProductVec[ v23 ] -  IdentityMatrix[4];
A29  = 2 * DyadicProductVec[ v29 ] -  IdentityMatrix[4];
A28  = 2 * DyadicProductVec[ v28 ] -  IdentityMatrix[4];


T=- MyTensorProduct[ A12, MyTensorProduct[ A16, MyTensorProduct[ A17,  A18]]]-
    MyTensorProduct[ A34, MyTensorProduct[ A45, MyTensorProduct[ A47,  A48]]]-
    MyTensorProduct[ A17, MyTensorProduct[ A37, MyTensorProduct[ A47,  A67]]]-
    MyTensorProduct[ A12, MyTensorProduct[ A23, MyTensorProduct[ A28,  A29]]]-
    MyTensorProduct[ A45, MyTensorProduct[ A56, MyTensorProduct[ A58,  A59]]]-
    MyTensorProduct[ A18, MyTensorProduct[ A28, MyTensorProduct[ A48,  A58]]]-
    MyTensorProduct[ A23, MyTensorProduct[ A34, MyTensorProduct[ A37,  A39]]]-
    MyTensorProduct[ A16, MyTensorProduct[ A56, MyTensorProduct[ A67,  A69]]]-
    MyTensorProduct[ A29, MyTensorProduct[ A39, MyTensorProduct[ A59,  A69]]];


Sort[N[ Eigenvalues[FullSimplify[T]] ]]

~~~~~~~~~~~~~~ Mathematica responds with

-6.94177, -6.67604, -6.33701, -6.28615, -6.23127, -6.16054, -6.03163, \
-5.96035, -5.93383, -5.84682, -5.73132, -5.69364, -5.56816, -5.51187, \
-5.41033, -5.37887, -5.30655, -5.19379, -5.16625, -5.14571, -5.10303, \
-5.05058, -4.94995, -4.88683, -4.81198, -4.76875, -4.64477, -4.59783, \
-4.51564, -4.46342, -4.44793, -4.36655, -4.33535, -4.26487, -4.24242, \
-4.18346, -4.11958, -4.05858, -4.00766, -3.94818, -3.91915, -3.86835, \
-3.83409, -3.77134, -3.7264, -3.68635, -3.63589, -3.59371, -3.54261, \
-3.48718, -3.47436, -3.4259, -3.35916, -3.35162, -3.29849, -3.24756, \
```

```
−3.23809, −3.18265, −3.14344, −3.09402, −3.07889, −3.03559, −3.02288, \
−2.98647, −2.88163, −2.84532, −2.80141, −2.76377, −2.72709, −2.67779, \
−2.65641, −2.64092, −2.5736, −2.53695, −2.48594, −2.46943, −2.42826, \
−2.40909, −2.3199, −2.27146, −2.26781, −2.23017, −2.19853, −2.14537, \
−2.1276, −2.1156, −2.08393, −2.02886, −2.01068, −1.95272, −1.90585, \
−1.8751, −1.81924, −1.80788, −1.77317, −1.71073, −1.67061, −1.61881, \
−1.58689, −1.56025, −1.52167, −1.47029, −1.43804, −1.41839, −1.39628, \
−1.33188, −1.2978, −1.26275, −1.24332, −1.17988, −1.16121, −1.12508, \
−1.06344, −1.04392, −0.981618, −0.9452, −0.93099, −0.902773, \
−0.866424, −0.847618, −0.797269, −0.749678, −0.718776, −0.667079, \
−0.655403, −0.621519, −0.563475, −0.535886, −0.505914, −0.488961, \
−0.477695, −0.438752, −0.413149, −0.385094, −0.329761, −0.313382, \
−0.267465, −0.251247, −0.186771, −0.162663, −0.135313, −0.115949, \
−0.0388241, −0.0285473, 0.0336107, 0.0472502, 0.0664514, 0.0818923, \
0.137393, 0.170784, 0.18296, 0.254586, 0.311604, 0.337846, 0.347853, \
0.351775, 0.395505, 0.422414, 0.481815, 0.515078, 0.57488, 0.600515, \
0.655748, 0.703362, 0.727865, 0.763394, 0.782482, 0.81889, 0.844406, \
0.888659, 0.920904, 1.00356, 1.02312, 1.03976, 1.08469, 1.1021, \
1.11609, 1.14654, 1.20192, 1.22992, 1.28624, 1.29287, 1.32196, \
1.36147, 1.43187, 1.52158, 1.5859, 1.61094, 1.62377, 1.66645, \
1.68222, 1.77266, 1.8082, 1.86793, 1.92219, 1.94603, 1.98741, \
2.04197, 2.06058, 2.12728, 2.16917, 2.20299, 2.20934, 2.2568, \
2.34362, 2.38008, 2.38999, 2.44382, 2.47456, 2.49679, 2.57822, \
2.62572, 2.63375, 2.67809, 2.73929, 2.81403, 2.82569, 2.87209, \
2.94084, 2.94773, 2.99356, 3.03768, 3.0484, 3.09975, 3.2194, 3.26743, \
3.2782, 3.30107, 3.41633, 3.43565, 3.49832, 3.62058, 3.6639, 3.7087, \
3.78394, 3.83644, 3.94999, 3.98744, 4.01948, 4.12536, 4.33452, \
4.37928, 4.42565, 4.47313, 4.53695, 4.71925, 4.84841, 4.90328, \
4.95742, 5.0169, 5.17123, 5.28471, 5.39555, 5.68376, 5.78503, 6.023}
```

---

[1] K. Fukuda, *cdd and cddplus homepage, cddlib package cddlib-094h* (2000,2017), accessed on July 1st, 2017, URL http://www.inf.ethz.ch/personal/fukudak/cdd_home/.

[2] Free Software Foundation, *GMP, arithmetic without limitations, the GNU multiple precision arithmetic library gmp-6.1.2.tar.lz* (1991,2017), accessed on July 29th, 2017, URL https://gmplib.org/.

[3] W. R. Inc., *Mathematica, Version 11.1* (2017).
